# The Mystery of Black TiO_2_: Insights from Combined Surface Science and In Situ Electrochemical Methods

**DOI:** 10.1021/acsmaterialsau.1c00020

**Published:** 2021-08-03

**Authors:** Ádám Balog, Gergely F. Samu, Szabolcs Pető, Csaba Janáky

**Affiliations:** Department of Physical Chemistry and Materials Science, Interdisciplinary Excellence Centre, University of Szeged, Aradi Square 1, Szeged H-6720, Hungary

**Keywords:** TiO_2_, defect engineering, photocatalysis, solar fuels, photoelectrochemistry

## Abstract

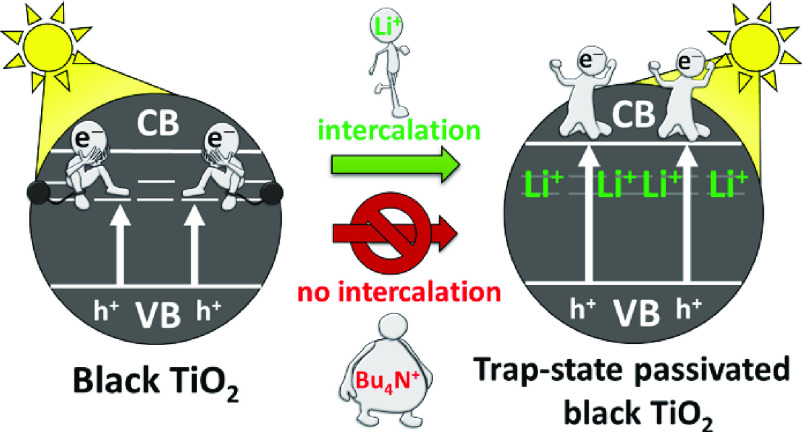

Titanium dioxide (TiO_2_) is often employed as a light absorber, electron-transporting material and catalyst in different energy and environmental applications. Heat treatment in a hydrogen atmosphere generates black TiO_2_ (b-TiO_2_), allowing better absorption of visible light, which placed this material in the forefront of research. At the same time, hydrogen treatment also introduces trap states, and the question of whether these states are beneficial or harmful is rather controversial and depends strongly on the application. We employed combined surface science and in situ electrochemical methods to scrutinize the effect of these states on the photoelectrochemical (PEC), electrocatalytic (EC), and charge storage properties of b-TiO_2_. Lower photocurrents were recorded with the increasing number of defect sites, but the EC and charge storage properties improved. We also found that the PEC properties can be enhanced by trap state passivation through Li^+^ ion intercalation in a two-step process. This passivation can only be achieved by utilizing small size cations in the electrolyte (Li^+^) but not with bulky ones (Bu_4_N^+^). The presented insights will help to resolve some of the controversies in the literature and also provide rational trap state engineering strategies.

## Introduction

Photoelectrochemical (PEC) methods hold the promise to produce valuable chemical products by combining the functions of solar cells and electrolyzers.^1^ After almost five decades of research on different semiconductors, the “wonder material” is still missing, which would pave the way toward the commercialization of PEC systems.^2,3^ TiO_2_ has been extensively studied because of its favorable band-edge positions to drive various relevant redox reactions (e.g., water oxidation).^4−7^ Further advantages of TiO_2_ are its low cost, abundance, and stability against photocorrosion.^8^ Besides these advantageous properties, there are two main issues that hinder its PEC application: a large bandgap energy (3.0–3.4 eV depending on the polymorph) that restricts the wavelength range of solar illumination TiO_2_ can harness and fast charge carrier recombination caused by the inherently high density of trap states.^9^ The first issue can be solved by introducing different dopants, which narrow the bandgap. The unintended side effect of this method, however, is the acceleration of the recombination process. Therefore, dopant-free procedures should be used to narrow the bandgap while overcoming the second limitation.^10^

During the past decade, a massive interest was devoted to hydrogen treatment induced modification of TiO_2_, which produces different defect states within the bandgap.^11,12^ This process results in a drastic color change, from which the name black TiO_2_ (b-TiO_2_) originates.^13^ The black color is the result of increased light absorption in the visible and near-infrared regime. There are examples in the literature where the photocatalytic^14,15^ and PEC^16−18^ activity of TiO_2_ were enhanced by this procedure. The improved PEC performance of b-TiO_2_ was explained by the interplay of five effects: (i) increased light absorption, (ii) improved charge carrier separation, (iii) decreased resistance of charge transport in the bulk, (iv) enhanced charge transfer at the semiconductor/electrolyte interface, and (v) suppressed recombination.^19^ An interesting observation was that the photocurrent enhancement is mainly caused by the improved photoactivity in the UV region.^18,20^ The narrowing of the bandgap did not automatically yield higher incident photon to charge carrier conversion (IPCE) values in the visible region. In fact, it had a negligible contribution to the total photocurrent.^21^ The photocurrent saturation, however, was achieved closer to the flatband potential, compared to its untreated counterpart, which indicates the better *catalytic* (i.e., *charge transfer*) performance of b-TiO_2_. Hydrogen treatment also increases the electron donor density by creating oxygen vacancies (Ti(III) sites) in the structure.^15,22^ These defects have low formation energies and are mainly responsible for enhancing the electrical conductivity of b-TiO_2_.^20^

Despite the extensive research on b-TiO_2_, there is still no clear evidence why it shows better performance in PEC and photocatalytic *oxidation* reactions, compared to its untreated white TiO_2_ (w-TiO_2_) counterpart, in certain reports.^18,20,23^ In these cases, the photogenerated holes are responsible for the PEC reaction, not the electrons that are present on the trap states. According to previous studies, the Ti(III) sites can bind O_2_ on the TiO_2_ surface, which enhances the band bending at the interface via charge-donating surface defects.^24^ At the same time, there are examples where the reaction efficiency is limited by the presence of such trap states, which facilitate the recombination process.^25^ To mitigate this problem, the traps should be passivated to keep the generated charge carriers separated, therefore suppressing recombination. One possible option for this is Li^+^ ion treatment prior to application; thus, the recombination pathway can be suppressed or even eliminated.^26,27^ In another example, the best photocatalytic activity was obtained for w-TiO_2_. After the reoxidation of the black sample (by heat treatment in air), the visible absorption (and also the black color) disappeared, and the electrical properties also reverted.^28^

These examples indicate that the effect of defects is controversial, and a better understanding of the structural and electronic changes that occur during the hydrogen treatment is necessary.^29^ According to certain transmission electron microscopy (TEM) studies, the enhanced catalytic properties of b-TiO_2_ are caused by a 1–2 nm thick disordered Ti_2_O_3_ surface shell surrounding a perfectly crystalline core.^30,31^ Most explanations for the narrower bandgap of b-TiO_2_ rely on the synergistic action between the oxygen vacancies and structural defects of nonspecific origin.^32^ However, the proposed outermost Ti_2_O_3_ is another semiconductor with a very narrow bandgap (∼0.1 eV), which might explain the phenomenon.^33,34^ This material is a stoichiometric compound that contains Ti(III) ions in a more stable form than oxygen-deficient TiO_2_. The problem with this interpretation is that in most cases the detection of Ti(III) in b-TiO_2_ is very difficult, which is surprising if the outermost layer contains only this oxidation state. These contradictions are summarized in Table S1. Finally, an interesting aspect is that most PEC studies on b-TiO_2_ were carried out exclusively using 1D TiO_2_ nanostructures, such as rutile nanowire arrays^17,20^ or nanotubes.^35^ In these configurations, the enhanced vertical charge transport ensured efficient charge extraction.^36^ In contrast, the photocatalytic studies mostly focus on nanoparticulate TiO_2_, where this charge transport avenue is absent. As P25 is the most widely studied among the TiO_2_-based photocatalysts, it is vital to understand how hydrogen treatment affects its properties.

In this paper, we present insights on the optoelectronic features of different nanoparticulate TiO_2_ electrodes deduced from in situ spectroelectrochemical methods and photoelectrochemical measurements. These results are complemented with X-ray and UV photoelectron spectroscopy studies. Based on these results, we analyze the influence of the trap states on the PEC, electrocatalytic (EC), and charge storage properties. Finally, we describe how to improve the PEC properties by passivating the defect sites by the incorporation of Li^+^ ions.

## Results and Discussion

### Characterization of the Differently Treated TiO_2_ Electrodes

**Figure 1:**
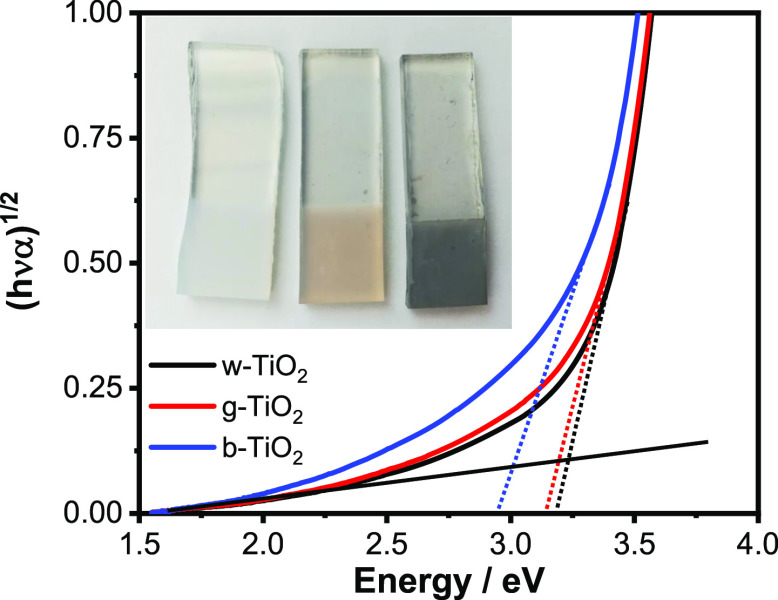
Bandgap energy determination of w-, g-, and b-TiO_2_ using Tauc analysis. The inset shows photographs of the respective FTO/TiO_2_ electrodes.

The Aeroxide P25 TiO_2_ nanoparticles were spray-coated on FTO substrates (with a geometrical surface area of 1 cm^2^) and annealed in air, argon, or hydrogen atmosphere in a tube furnace. After the heat treatment, the color of the electrodes changed (inset of Figure 1), and based on this color, we denote them as white (w-), gray (g-), and b-TiO_2_, respectively. All samples showed strong UV absorption (Figure S1) related to the electronic transition from the valence band (VB) to the conduction band (CB). The w- and g-TiO_2_ samples showed small absorption in the visible region, which is likely caused by light scattering of the layers. Compared to these, b-TiO_2_ showed an increased visible light absorption, associated with the transition from the VB to different midgap states such as the Ti(III) states (i.e., oxygen vacancies).^32^ The w-TiO_2_ also contains certain amounts of these states (discussed later); however, this does not have a marked influence on its absorption characteristics. There are examples in the literature where absorption features also appeared in the near-infrared region, attributed to the excitation from the Ti(III) levels to the CB.^38^ In our case, we could not observe such features. The bandgaps, which were calculated from Tauc plots derived for an indirect transition (Figure 1), slightly decreased with the darkening of the color. This is also in correlation with the increasing reducing ability of the heat-treatment atmosphere. Indirect bandgap values of 3.25 eV for w-TiO_2_, 3.20 eV for g-TiO_2_, and 3.00 eV for b-TiO_2_ were determined.

XPS measurements were carried out to evaluate the chemical composition of the surface of the three differently treated TiO_2_ samples. The survey scans showed identical chemical species on the surface of the samples (Figure S2). Only one chemical component was necessary to fit the high-resolution Ti 2p spectra in all cases (Figure S3A and Table S3). This corresponds well to the binding energy of Ti(IV) in TiO_2_ materials (458.66 eV with a peak separation of Δ*E* = 5.66 eV).^39^ The presence of surface Ti(III) species (2p_3/2_ at 457.13 eV) was not observed for any of the samples. The determined binding energy of Ti(IV) was similar in the case of the w- and g-TiO_2_ samples (Table S3). In the case of b-TiO_2_, however, a significant shift was observed toward higher binding energies. This alteration in the peak position can be assigned to structural distortions on the surface of the b-TiO_2_ sample (as discussed later).^40^ The resolved O 1s region was also very similar for all samples (Figure S3B). The oxygen on the surface can be assigned to the Ti–O bond at 529.95 ± 0.13 eV and surface Ti–OH groups at 531.58 ± 0.20 eV.^41^

To evaluate the effect of heat treatment in the different atmospheres on the crystallinity, XRD measurements were carried out (Figure S4). Rietveld refinement identified anatase and rutile phases in a 3:1 ratio in all cases, which agrees with the composition of P25 Aeroxide TiO_2_. The heat treatment had a negligible (if any) effect on the chemical composition of the films, in line with other reports.^32,42−45^ This signals that either (i) limited conversion to the suboxide phase occurred and that less than 1% Ti(III) formed on the surface (i.e., below the detection limit of XPS)^43^ or (ii) a defective disordered surface was obtained, with a stoichiometric composition.^32^ To reveal the subsurface composition, Ar^+^ bombardment was carried out, to etch the surface of the w- and b-TiO_2_ samples (Figure S5A,B). Note that high-energy Ar^+^ bombardment can also reduce Ti(IV), yielding Ti(III) in the samples^46,47^ as it is apparent for the w-TiO_2_ case (Figure S5A). Importantly, in the case of b-TiO_2_, an increased amount of Ti(III) was detected after bombardment (compared to w-TiO_2_), and also, the presence of Ti(II) could be identified after identical bombardment conditions (Figure S5B). This signals that b-TiO_2_ either: (i) inherently contains more Ti(III)/Ti(II) sites beneath its surface or that (ii) it is more susceptible to Ar^+^ bombardment than its w-TiO_2_ counterpart. Notably, electron paramagnetic resonance measurements on b-TiO_2_ materials synthesized under similar conditions show a strong paramagnetic signal, which can be attributed to the formation of Ti(III) centers.^12^

**Figure 2:**
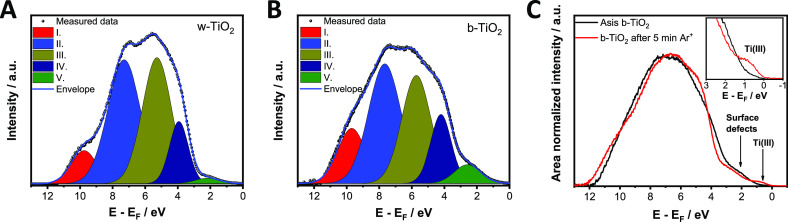
Background subtracted and deconvoluted He(I) ultraviolet photoelectron spectra of (A) w- and (B) b-TiO_2_ samples. (C) Background subtracted and area normalized He(I) ultraviolet photoelectron spectra of b-TiO_2_ before (black) and after (red) 5 min of Ar^+^ bombardment. Inset shows magnified region where the Ti(III) state is observable.

To determine the position and the electron density of the VB, He(I) ultraviolet photoelectron spectroscopy (UPS) measurements were performed for w- and b-TiO_2_ electrodes (Figure S6). The VB edge region shows two distinct features: a steep and a shallow cutoff. The VB edge calculated from the onset of the steep region gave similar values for w-TiO_2_ (−7.26 eV vs vacuum) and b-TiO_2_(−7.28 eV vs vacuum), which is in line with earlier reports.^20,48^ The shallow region can be assigned to the electron density within the bandgap. The background subtracted UPS spectra of the VB region were deconvoluted into five components (Figure 2 and Table S2).^48^ This is in line with the results from density functional theory (DFT) calculations.^49^ The state at the highest binding energy (I) corresponds to surface −OH species, which has a greater contribution in the case of b-TiO_2_. The following three (II–IV) components are attributed to hybridized Ti 2p and O 2p orbitals, while the state (V) at the lowest binding energy corresponds to electron density related to surface defects. Importantly, the area of state V is 3 times larger in the case of b-TiO_2_ compared to w-TiO_2_. Furthermore, no features related to Ti(III) (3d) states from either oxygen vacancies or Ti(III) interstitials can be observed, which would be located between 0 and 2 eV binding energy, situated below the CB edge.^50,51^ As UPS is considered more surface sensitive (2–5 nm penetration depth) than XPS, this also points toward the disordered-surface/crystalline-core structure where eventual Ti(III) moieties are located in the crystalline core, residing below the information depth of both XPS and UPS. This is not surprising, because exposure of the as-synthesized b-TiO_2_ samples (with potential Ti(III) content at the surface) to oxygen after the synthesis (even at room temperature) can convert the outmost surface to stoichiometric TiO_2_ leaving a disordered structure behind.^32^ This lattice disorder can also result in the formation of surface defects (see discussion later), instead of forming discrete energy levels (as in the case of Ti(III) states), which results in a continuum electron density starting from the VB edge up to the CB edge (band tail states).^14,32^ The observed tailing on the UPS spectra can be the sign of this disordered structure of the surface of b-TiO_2_. Previous studies also confirm the presence of the disordered shell by high-resolution TEM measurements.^14^ To visualize the difference between surface defects and Ti(III) states, the UPS of b-TiO_2_ was recorded before and after Ar^+^ bombardment (Figure 2C). With the emergence of Ti(III) upon the bombardment (see also Figure S5), a well-defined state arises within the bandgap at 0.78 eV (translating to an energy level of −5.31 eV vs vacuum).

### Electrochemical Properties of the Different TiO_2_ Electrodes in Aqueous Electrolyte

**Figure 3:**
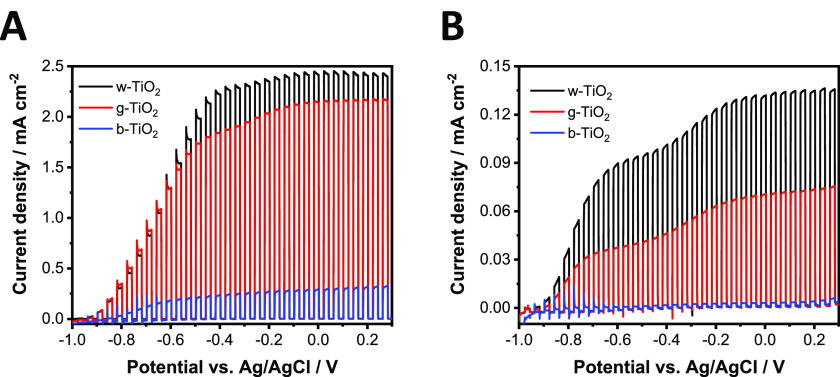
Photovoltammograms of w-, g-, and b-TiO_2_ electrodes. The measurements were recorded in argon-saturated 0.1 mol dm^–3^ Na_2_SO_3_ electrolyte in water, using (A) a UV lamp and (B) a solar simulator (AM1.5) as the light source operated at 100 mW cm^–2^. The sweep rate was kept at 2 mV s^–1^, while the light-chopping frequency was 0.10 Hz.

PEC measurements were carried out to investigate the photoactivity of the three different TiO_2_ electrodes using either a UV lamp (Figure 3A) or a solar simulator (Figure 3B). For these initial experiments, aqueous solutions were employed: 0.1 M Na_2_SO_4_ (i.e., without hole scavenger, Figure S7) and 0.1 M Na_2_SO_3_ (i.e., with hole scavenger, Figure 3). Note that in the latter case, Na_2_SO_3_ functions as both the conducting salt and hole scavenger species. According to the literature, we expected to achieve the highest photocurrent in the case of b-TiO_2_. Surprisingly, in both electrolytes, the largest photocurrent was recorded for the w-TiO_2_ electrode. The pronounced difference among the electrodes, even in the presence of a hole scavenger, shows that the photocurrent is not limited by the kinetics of the charge transfer reaction. This points toward excessive trapping of electrons and subsequent recombination of charge carriers in the case of b-TiO_2_, which is in turn the limiting factor of the photocurrent. The difference among the samples became even more pronounced when we changed the illumination from UV light (Figure 3A) to simulated sunlight (Figure 3B), signaling that the b-TiO_2_ sample poorly utilizes the visible portion of sunlight.

**Figure 4:**
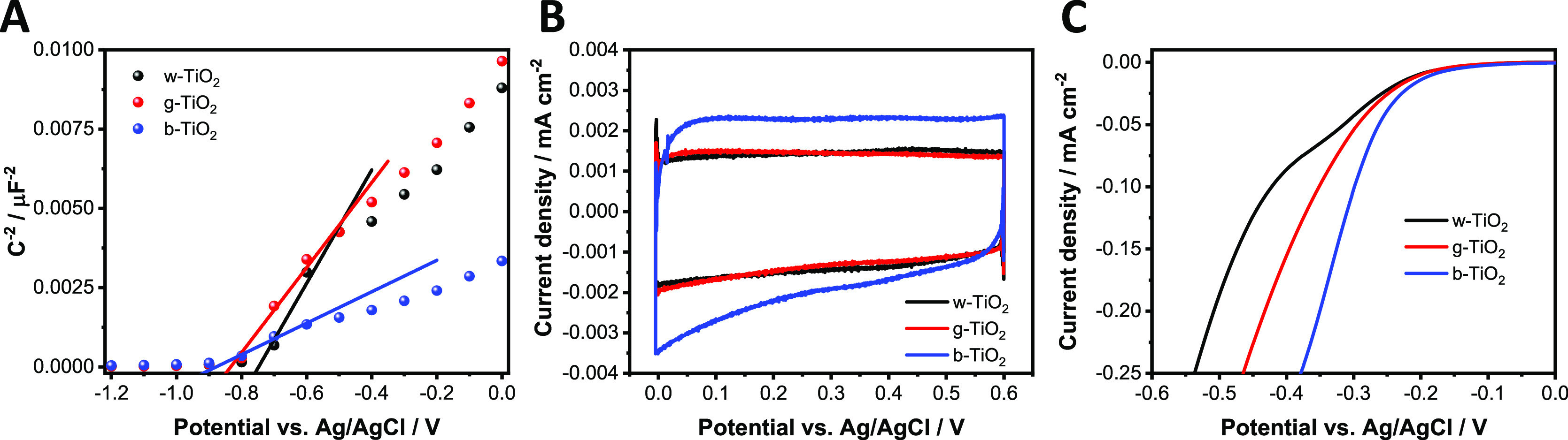
(A) Mott–Schottky plots at a 1 kHz frequency and (B) cyclic voltammetry in argon-saturated 0.1 mol dm^–3^ Na_2_SO_4_ solution with a sweep rate of 100 mV s^–1^. (C) Linear voltammetry in oxygen-saturated 0.1 mol dm^–3^ Na_2_SO_4_ solution with a sweep rate of 5 mV s^–1^.

The population of trap states with electrons affects the electrical properties of semiconductors, and this can be tracked by Electrochemical Impedance Spectroscopy (EIS, Figure S8).^52,53^ To determine the donor density, Mott–Schottky analysis was carried out (Figure 4A). The steepest slope was found for w-TiO_2_, while the smallest one was found for b-TiO_2_. The numbers of calculated donors were as follows: 4.6 × 10^19^ cm^–3^ (w-TiO_2_) < 6.1 × 10^19^ cm^–3^ (g-TiO_2_) ≪ 1.64 × 10^20^ cm^–3^ (b-TiO_2_). Therefore, the density of donors increases in parallel with the darkening of the color. Furthermore, the amount of midgap states (sum of surface defects and Ti(III) states) shows anticorrelation with the maximum achievable photocurrent for the samples (Figure 3). The excited electrons can be trapped at these states (instead of reaching the CB of TiO_2_), and therefore, they are not involved in the generation of the photocurrent. This notion was further confirmed by the PEC measurements using simulated sunlight (Figure 3B). In this case, a smaller portion of the light can excite electrons from the VB to the CB; therefore, most of the electrons will be trapped without generating photocurrent (i.e., the amount of trapped electrons represents a larger fraction of the total photogenerated electrons).

Cyclic and linear sweep voltammetry (LSV) scans were recorded to probe the charge storage (Figure 4B) and EC (Figure 4C) behavior of the different TiO_2_ electrodes. The enhancement in the capacitance of b-TiO_2_ compared to the g- and w-TiO_2_ (Figure 4B) can be attributed to two factors: (i) increased charge carrier density and (ii) increased density of surface hydroxyl groups that provides pseudocapacitive properties.^54^ As XPS revealed a similar surface composition for all samples (Figure S3), the first reason is more likely. The EC properties toward the oxygen reduction reaction (ORR) improved with the increase in the electron density of midgap states (Figure 4C). Interestingly, the onset potential of ORR coincides with the potential where the shape of the Bode plot started to change for each TiO_2_ sample (Figure S8). This suggests that instead of the CB, the filled midgap states play an important role in the ORR, as the onset potential for ORR is more positive than the flatband potential (which is around −0.8 V vs Ag/AgCl) (Figure 4A). We found, however, that this beneficial effect cannot be harnessed during PEC operation. This might be because of the sluggish electron extraction from the subsurface Ti(III) states that ultimately limits the photocurrent even when the hole extraction is accelerated (i.e., by using hole scavengers).

### Passivation of Trap States in the Different TiO_2_ Electrodes in Nonaqueous Electrolyte

**Figure 5:**
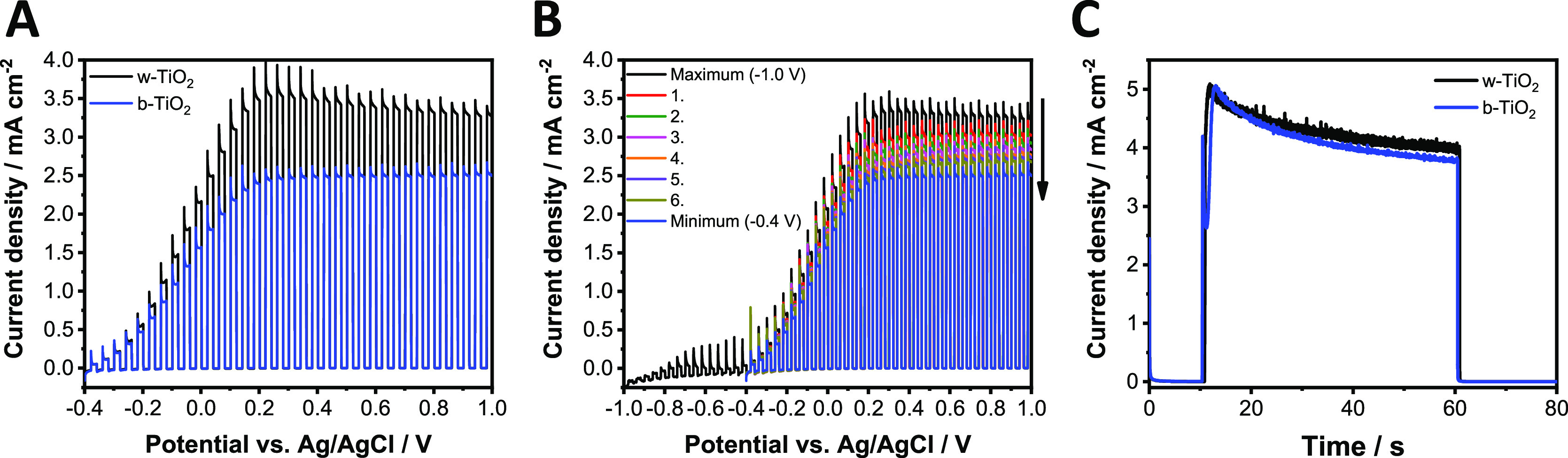
(A) Photovoltammograms of w- and b-TiO_2_ electrodes. (B) Photovoltammograms measured one after another for hydrogen-treated TiO_2_. The sweep rate was kept at 2 mV s^–1^, while the light-chopping frequency was 0.10 Hz. (C) Potentiostatic measurements of w- and b-TiO_2_ at +0.6 V after a pretreatment at −1.0 V for 1 min. The experiments were recorded in argon-saturated 1 mol dm^–3^ LiClO_4_ electrolyte in acetonitrile, using a UV lamp as the light source operated at 100 mW cm^–2^.

To passivate the trap states and improve the PEC properties, we performed Li^+^ incorporation/intercalation experiments.^26,27^ For the sake of simplicity, we only examined the w- and b-TiO_2_ electrodes in acetonitrile (ACN)-based solutions with LiClO_4_ as the electrolyte and methanol as the hole scavenger. Application of a nonaqueous solutions is advantageous, because sufficiently negative potentials can be employed to investigate the electrochemical reduction of TiO_2_ without any gas evolution (i.e., hydrogen evolution reaction). Importantly, the change in the media had no effect on the trend in the observed photocurrent values (Figure 5A). We found that the passivation of trap states in b-TiO_2_ is a two-step process. Interestingly, these passivation steps start well before the electrochemical reduction of TiO_2_ (see discussion later). This signals that only trap states, formed during the reductive thermal treatment, participated in these processes. The first step is a slow spontaneous uptake of Li^+^ from the electrolyte, which happens when the electrode is immersed in the solution (i.e., even without applied potential, Figure S9A). During this process, a gradual increase of the maximum photocurrent was observed. The Li^+^ uptake can be accelerated by applying a mild negative potential of −0.4 V (Figure S9B). This surface related process also changes the shape of the photocurrent transients. The initial slow rise of the photocurrent for the untreated b-TiO_2_ sample signals that electron trapping occurs, which limits the achievable anodic steady state photocurrent. As Li^+^ is incorporated, this gradually changes to a recombination limited photocurrent response, further proving effective defect passivation at the surface.^55^

This first passivation step, however, cannot increase the photocurrent over 2.5 mA cm^–2^, which still lags behind the performance of w-TiO_2_ electrodes. The trap passivation can also be observed on the photovoltammograms (Figure 5A) when the measurements were started from −0.4 V, which is not negative enough to initiate the second stage of trap state passivation in b-TiO_2_ (i.e., the passivation of subsurface Ti(III) states). Interestingly, when the photovoltammograms were recorded from −1.0 V, a further photocurrent increase was seen for b-TiO_2_, which ultimately reached the value recorded for w-TiO_2_ (Figure 5B). This can be considered as the second step of trap state passivation, which can only be achieved through proper electrochemical polarization. Notably, the photocurrent of w-TiO_2_ electrode was not influenced by the start potential (Figure S10). Finally, if the trap states were fully passivated (by a pretreatment at −1.0 V for 1 min), and the photocurrent transient was recorded *immediately* after this treatment, similarly high photocurrents were detected for the b- and w-TiO_2_ electrodes (Figure 5C). This is because applying potentials more positive than the open circuit potential (OCP) slowly drives Li^+^ into the electrolyte and reactivates the Ti(III) states during the LSV scans. This process is absent here; therefore, higher photocurrents are measured.

IPCE measurements were carried out to study how the two differently treated TiO_2_ electrodes utilize incident light (Figure S11). Without passivation, b-TiO_2_ shows low IPCE values in both the UV and visible range and an identical bandgap energy to its w-TiO_2_ counterpart (Figure S11A). After PEC measurements were performed from −1.0 V (trap state passivation), the IPCE values equalize (Figure S11B). Closer inspection of the IPCE curves of b-TiO_2_ reveal a slight visible light response, which confirms that the excitation to the trap states is possible to a small extent (Figure S11C). After trap state passivation, the visible light utilization of b-TiO_2_ gradually decreases. This is coupled with the shift in the minimum excitation energy required to produce any measurable photocurrent to lower wavelengths.

**Figure 6:**
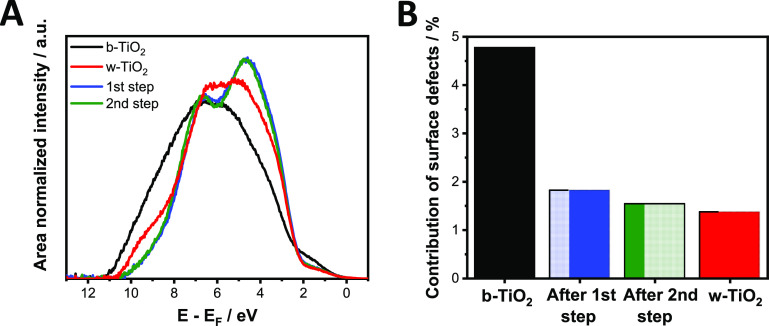
(A) Background subtracted and area normalized He(I) ultraviolet photoelectron spectra of w- (red) and b-TiO_2_ before (black) and after the first step (blue) and after the second step (green) of trap state passivation in a 1 mol dm^–3^ LiClO_4_ electrolyte in acetonitrile. (B) The contribution of surface defect states to the overall UPS spectra obtained after deconvolution of the background subtracted data.

To monitor the effect of Li^+^ incorporation on the surface composition, ex situ XPS and UPS measurements were carried out. The b-TiO_2_ samples were removed from the electrolyte after either the first or the second step of trap state passivation. In both cases, XPS measurements revealed that the binding energy of Ti(IV) 2p core line approached the values recorded for w-TiO_2_ samples (Figure S12, Table S3). This signals the partial reorganization of the surface disorder that was created by the hydrogen treatment of the samples (during the first passivation step already). This process also manifests in the UPS spectra (Figure 6A). The VB tailing caused by the presence of surface defects decreased already after the first passivation step. The overall contribution of the surface defect state halved from its original value in b-TiO_2_ (Figure 6B). Furthermore, a marked increase in the electron density in states III (Ti 3d t_2_g O 2p hybrid orbital) and IV (O 2p nonbonding orbital) is also apparent (Figure 6A).^56^ This larger electron density near the VB edge shows the stronger overlap between molecular orbitals, where O 2p orbitals are participating. This signals that these molecular orbitals are situated closer to each other in the reformed lattice.^57^ There was no observable difference between the two passivation steps, as the second stage affects the deeper regions of the samples where the surface defect states are already passivated.

To check whether the Ti(III) state passivation is permanent for b-TiO_2_, we recorded the first photovoltammogram from −1.0 V and all subsequent scans from −0.4 V (Figure 5B). We found that Li^+^ ions were gradually removed from these states (as seen in Figure 5B). During these PEC experiments, the potential was scanned with a slow sweep rate, which ensured slow but continuous depletion of Li^+^ ions from the Ti(III) defects. We also performed potentiostatic experiments at different potentials to study the removal of Li^+^ ions from b-TiO_2_ (Figure S13). Before each measurement, the Ti(III) sites were passivated at −1.0 V for 1 min. We found that these states remain passivated unless a potential more positive than the OCP (∼−0.2 V) is applied to the electrodes before the photocurrent is measured (Figure S13A). When this potential is applied, the photocurrent decreases exponentially as more time is elapsed (Figure S13A). Furthermore, the photocurrent cannot be decreased below the value than what is achieved with the spontaneous surface defect passivation. Interestingly, the achieved photocurrent in this second step depends only on the time of this treatment and is independent from the applied potential (Figure S13B).

**Scheme 1:**
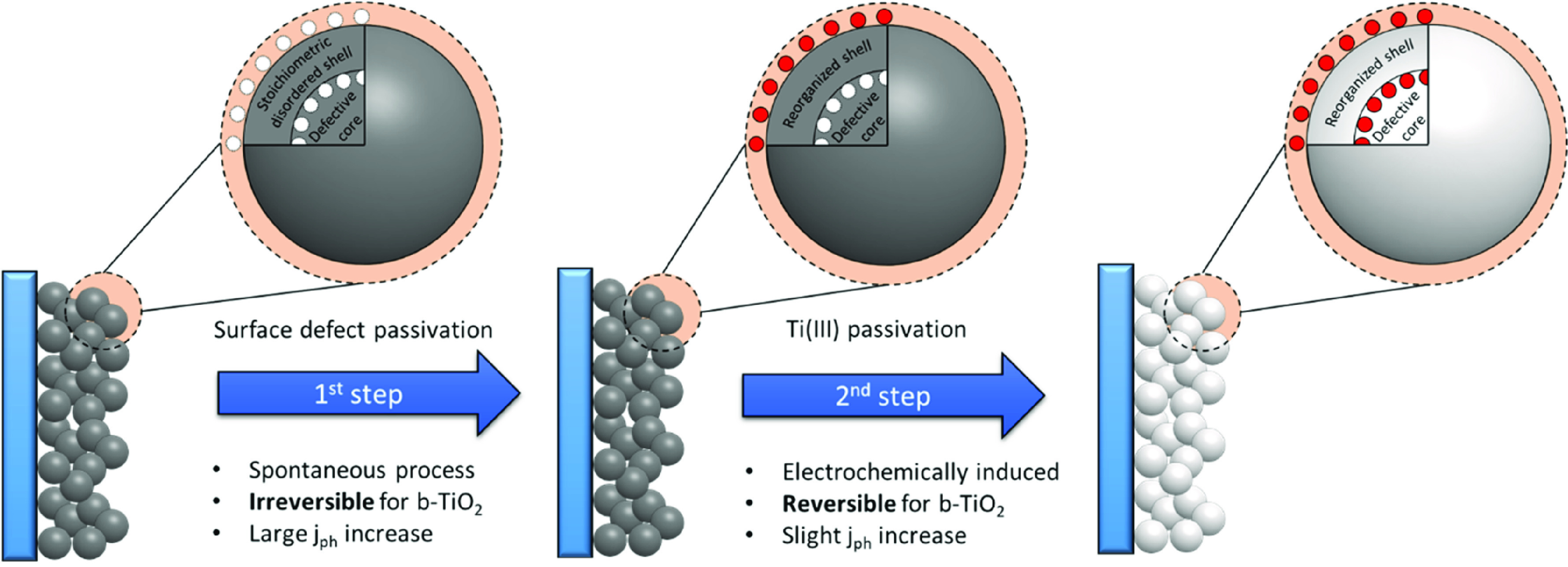
Depiction of the Two-Step Trap State Passivation of Electrodes Consisting of Nanoparticulate b-TiO_2_ Materials in Li^+^-Containing Media

We propose that during the first step, trap states closer to the surface of the TiO_2_ particles (surface defects, related to the disordered structure) are passivated with Li^+^(shown in Scheme 1). This process was found to be spontaneous (and irreversible) and caused a more than 4-fold increase in the maximum photocurrent of b-TiO_2_ sample (likely tied to the reorganization of the disordered surface). When a negative polarization is applied, the Ti(III) states (located beneath the surface of the particles) can also be passivated, resulting in a smaller photocurrent increase. We found that the prolonged second passivation step also results in the loss of the black color of the samples, which points toward the complete removal of the Ti(III) defects in b-TiO_2_.

To prove that Li^+^ ions are incorporated into the TiO_2_ layers during the trap state passivation steps, ion chromatography experiments were carried out. In the first step, trap state passivation was carried out by applying −1.0 V potential for 5 min in LiClO_4_-containing ACN solution. After the electrodes were removed from the electrolyte, they were carefully washed with pure ACN and deionized water. The electrolyte was changed to 2 cm^3^ of aqueous 0.01 mol dm^–3^ KCl, and the removal of Li^+^ ions was performed by applying a potential of +0.6 V for 30 min. The peak corresponding to Li^+^ ions was detected on the chromatograms (Figure S14). The total area of this peak was 3 times smaller in the case of w-TiO_2_ compared to b-TiO_2_, which is in good correlation with the relative donor densities (1.64 × 10^20^ cm^–3^ (b-TiO_2_)/4.6 × 10^19^ cm^–3^ (w-TiO_2_) = 3.56) determined from Mott–Schottky measurements (Figure 4A).

Besides the photocurrent values, the capacitance can also be altered by the electrochemical treatment. This is also reflected in the capacitance values determined from cyclic voltammetry (Figure S15), which shows lower capacitance values for the b-TiO_2_ samples after the pretreatment at potentials more negative than −0.4 V. This signals the decrease in the number of donor states during Li^+^ incorporation. This statement was further proved by Mott–Schottky experiments (Figure S16). After Li^+^ incorporation, almost identical slopes were found for the two materials, which signals that the number of donors became equal during the measurement. As shown previously (Figure S13A), the passivation and especially the removal of Li^+^ ions is not immediate. Repeating the Mott–Schottky experiment three times using the same sample, a continuous decrease of the donor density was found in the case of b-TiO_2_, while it remained unchanged in the case of w-TiO_2_ (Figure S17).

**Figure 7:**
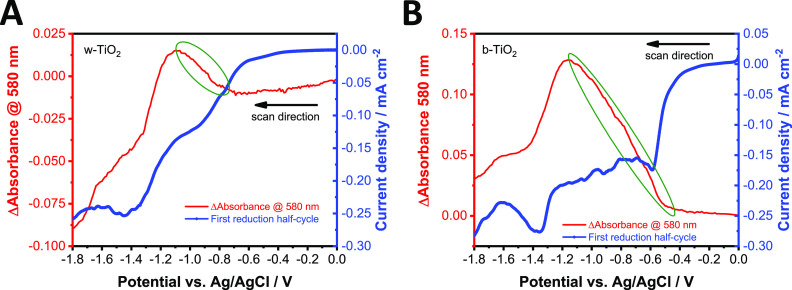
Spectroelectrochemical data, recorded for w- and b-TiO_2_ electrodes. The first reduction half-cycles of (A) w- and (B) b-TiO_2_ are plotted together with the Δ*A* at 580 nm. The experiments were recorded in oxygen-saturated 1.0 mol dm^–3^ LiClO_4_ electrolyte in acetonitrile with a sweep rate of 1 mV s^–1^. The green circles highlight the change of the absorbance during the electron injection to the trap states.

Spectroelectrochemical experiments were carried out to monitor the population of trap states of the two TiO_2_ electrodes. The potential was scanned from 0.0 to −1.8 V with a sweep rate of 1 mV s^–1^. To visualize the changes in the optical properties, the first (Figure 7A,B) and second (Figure S18A,B) reduction half-cycles were plotted together with the Δabsorbance measured at 580 nm. Two reduction peaks can be observed in all cases, one of which appears at the same potential with an onset of ∼−1.2 V, regardless of the sample type and the number of half-cycles. This peak corresponds to the *x*Li^+^ + Ti(IV)O_2_ + *x*e^–^ → Li_*x*_Ti(III)O_2_ reaction, which is the electrochemical reduction of TiO_2_.^58^ At potentials more negative than −1.2 V, a broad absorption appears in the visible and near-infrared region of the UV–vis spectrum (Figure S19B,D). This indicates that free electrons are present in the accumulation layer. Accumulation of electrons in the CB is accompanied by the bleaching of interband transitions near the absorption threshold (Figure S19B,D).^59^ The other peak in the half-cycle, which is more pronounced in the case of b-TiO_2_, is the sum of ORR and trap filling current.^60^ If we compare the first reduction peak of the two different TiO_2_ electrodes during the first half-cycle (Figure 7A,B), the onset of the cathodic current is shifted to more positive potentials in the case of b-TiO_2_, compared to w-TiO_2_. The accompanying absorbance increase (Figure S19A,C) is related to an increase in electron population at the Ti(III) states (second passivation step).^61^ The change in the absorbance (and also the injection current) is much higher in the case of b-TiO_2_, which further confirms the presence of a more defective structure. When comparing the second cathodic half-cycles performed immediately afterward, the onset of the first peak shifts toward more negative potentials (Figure S18A,B). The change in the absorbance is also less pronounced, to an extent, where it completely disappears in the case of w-TiO_2_. This signals that a large portion of the Ti(III) sites are eliminated during the first cycle; thus, in the second half-cycle, only the remaining defects can be filled with electrons.

**Figure 8:**
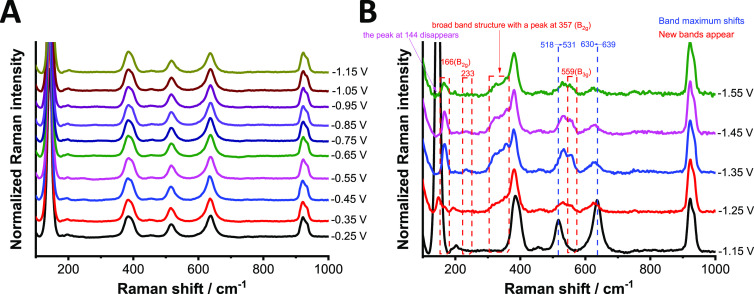
In situ Raman spectroelectrochemistry of w-TiO_2_ in the range between (A) −0.25 to −1.15 V and (B) −1.15 to −1.55 V recorded in 1 mol dm^–3^ LiClO_4_ electrolyte in acetonitrile.

To investigate the changes in the structural features of TiO_2_ as a function of potential, in situ Raman spectroelectrochemistry was performed (Figures 8 and S20). During these measurements, a Raman spectrum was recorded at every 100 mV in the range between −0.25 to −1.55 V. Until the onset of the electrochemical reduction current of TiO_2_ (black dotted line in Figure S20A), which is associated with the electrochemical formation of Ti(III) sites and stabilization by Li^+^ ions, the Raman spectra were unaltered for both materials (Figures 8A and S20C). This shows that the previous absorbance increase in the UV–vis spectra can be attributed to the change in the population of Ti(III) sites, which were produced during the heat treatment, as there are no structural changes between −0.25 and −1.15 V. When the potential was further decreased, several changes were detected in the Raman spectra (Figures 8B and S20D). These changes indicate the phase transition of TiO_2_ from tetragonal to orthorhombic accompanying Li^+^ ion intercalation (see the detailed description in the Supporting Information).

### Construction of the Band Energy Diagrams

**Figure 9:**
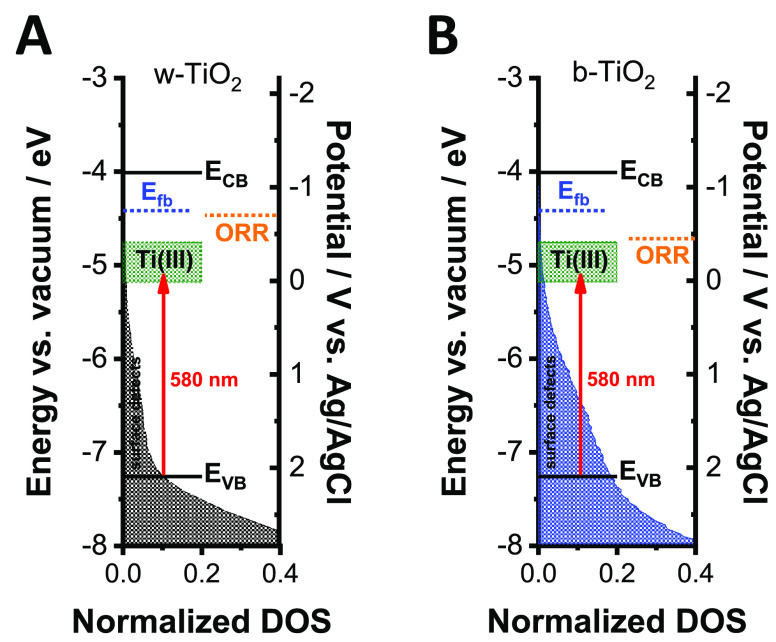
Band diagram of (A) w- and (B) b-TiO_2_ electrodes with the position of Ti(III) states (green boxes). The flatband potential (blue dotted line), the onset of the ORR (orange dotted line), and the optical transition (red arrow), which is responsible for the spectral changes, are also marked on the band diagram. The filled areas represent the relative density of states at different energy levels.

Taking the results of all optoelectronic studies as a whole, the band diagram of TiO_2_ was constructed for the w-TiO_2_ and b-TiO_2_ electrodes (Figure 9). The UPS data was used as the framework, while the CB position was calculated (−4.01 eV vs vacuum) using the optical bandgap of w-TiO_2_ (3.25 eV) obtained from Tauc analysis. The filled areas represent the relative density of surface defects at different energy levels. The flatband potential (blue dotted line), the onset of the ORR (orange dotted line), and the optical transition (red arrow), which is responsible for the spectral changes in the UV–vis spectra, are also marked on the band diagram. The green areas show the Ti(III) defects, which are 0.75 and 1.18 eV below the CB edge according to the literature.^62^ The position of these states was also confirmed by the UPS measurement after 5 min of Ar^+^ bombardment (Figure 2C). In principle, the electrons can reach the TiO_2_/electrolyte interface only at more negative potentials than the flatband (∼−0.8 V). This was the situation in the case of w-TiO_2_ (Figure 9A); however, the onset of the ORR on b-TiO_2_ can be observed even before the flatband potential (Figure 9B). This signals that the disordered layer at the surface of b-TiO_2_ can act as a catalyst and lower the overpotential of the ORR. However, this effect can only be achieved after the Ti(III) states are filled. As the disordered-surface layer is absent in the case of w-TiO_2_, the ORR starts at more negative potentials on w-TiO_2_. This can rationalize the higher catalytic activity of b-TiO_2_ compared to its white counterpart.

The optical changes start exactly from the potential (−0.45 V in the case of b-TiO_2_ and −0.7 V in the case of w-TiO_2_) where the ORR peak appears in the cyclic voltammograms. The absorbance increases in a broad wavelength range with a maximum at ∼580 nm (2.14 eV) (Figure S19A,C), which corresponds to the excitation from the VB to the Ti(III) states. The appearance of the broad absorption band is not surprising, as there is significant electron population both above (surface defects) and below the VB edge. This was confirmed by suface photovoltage spectroscopy (SPS) measurements, which determined 1.61 eV (w-TiO_2_) and 1.40 eV (b-TiO_2_) as the lowest energies that are needed to create electron–hole pairs in the different TiO_2_ electrodes (Figure S21). As the ORR starts to deplete electrons from the filled states, the path to excite electrons from the VB to the Ti(III) defects becomes more pronounced, signaled by the increase in the absorbance at ∼580 nm.

### Effect of the Cation Size on the Trap State Passivation Process

**Figure 10:**
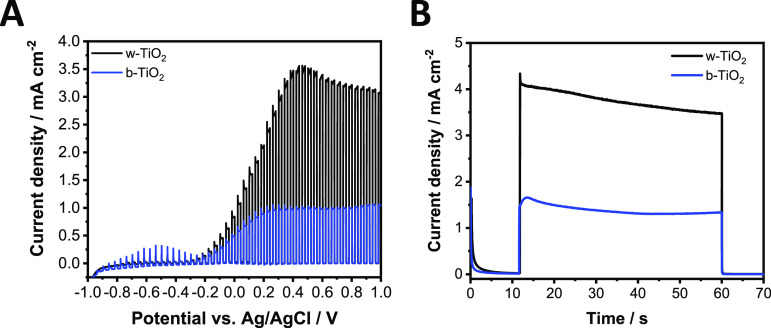
(A) Photovoltammograms of w- and b-TiO_2_ electrodes. The sweep rate was kept at 2 mV s^–1^, while the light-chopping frequency was 0.10 Hz. (B) Potentiostatic measurements of w- and b-TiO_2_ at +0.6 V after a pretreatment at −1.0 V for a minute. A UV lamp was used as the light source operated at 100 mW cm^–2^. All the experiments were recorded in argon-saturated 1 mol dm^–3^ Bu_4_NClO_4_ electrolyte in acetonitrile.

To prove that the Li^+^ ions are responsible for the changes in the optoelectronic properties, we changed the Li^+^ cations in the electrolyte to bulky cations, namely to Bu_4_N^+^. In this case, the intercalation is not possible, so we could scrutinize the inherent electrochemical properties (without trap state passivation) of the two different TiO_2_ films. The difference between the photocurrents was larger compared to the LiClO_4_ electrolyte, and these values could not be improved by electron injection (Figures 10 and S22). Importantly, both previously described trap state passivation steps were absent in this electrolyte.

### Effect of the Heat Treatment on the Electrocatalytic Activity

**Figure 11:**
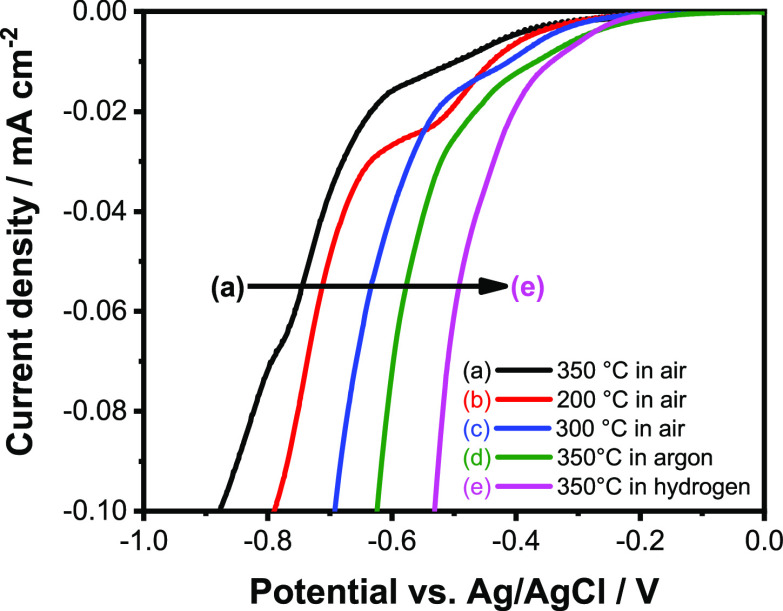
Linear voltammetry in oxygen-saturated 1 mol dm^–3^ LiClO_4_ electrolyte in acetonitrile with a sweep rate of 1 mV s^–1^.

Finally, we demonstrate that the number of defects in TiO_2_ can be controlled by the conditions of the heat treatment (temperature, atmosphere, and time). The onset of the ORR current can be used as an indicator of such defect formation (Figure 11). Notably, there is an intricate interplay between these defects and the crystallinity of the samples. At low annealing temperatures (200–300 °C in air atmosphere), the number of defects in the material increases with temperature, which manifests in the shift of the onset of the ORR current to more positive potentials. At ∼350 °C, however, the crystallinity of the samples becomes complete (the temperature is high enough to achieve the completely crystalline state during that 2 h heat treatment), causing a sudden negative shift in the onset potential. Further variation in the defect density can only be achieved by changing the atmosphere to a more reductive one (i.e., H_2_).

## Conclusions

The light absorption of TiO_2_ can be significantly altered using heat treatment in different atmospheres. Even though b-TiO_2_ prepared by hydrogen treatment possesses increased visible light absorption, it performs poorly in PEC oxidation processes. The highest photocurrent can be achieved with w-TiO_2_, which contains the least amount of trap states. The poor performance of b-TiO_2_ can be explained by the deleterious effect of defect sites, namely electron accumulation on surface defects and subsurface Ti(III) states upon light excitation. These trapped electrons cannot participate in photocurrent generation processes. At the same time, defect-rich b-TiO_2_ shows enhanced EC and capacitive properties compared to its w-TiO_2_ counterpart. The formed trap states (surface defects and subsurface Ti(III) sites) can be passivated by Li^+^, which results in increased photocurrents, ultimately leading to identical properties of the photoelectrodes, irrespective of the synthetic history. With the elimination of defects, the capacitance and the ORR ability also equalize, which means that a choice must be made.

In the passivation process, the size of the cation that is present in the electrolyte is also important. When a defect-rich semiconductor is intended, bulky cations (e.g., Bu_4_N^+^) must be used to prevent the intercalation process, therefore preserving trap states within the material. However, in the case of small cations (e.g., Li^+^), this intercalation step can take place even spontaneously without applying electrical bias after the electrode is immersed in the electrolyte. We hope that by identifying two different trap states as well as their contribution to the optoelectronic properties of b-TiO_2_ will help to resolve some of the controversies in the literature. As we have shown that a spontaneous passivation step is responsible for the majority of the photocurrent increase (but not the color change), notably different properties can be measured for similarly black TiO_2_ samples with different chemical/synthetic history.

## Experimental Section

### Materials

For the preparation of the spray-coating suspension, Aeroxide P25 TiO_2_ (Evonik) was dispersed in absolute ethanol (VWR, 100%). The FTO (fluoride doped tin oxide, Sigma-Aldrich, ∼7 Ω cm^–2^) covered glass substrates were cleaned with acetone (VWR, 100%), 2-propanol (VWR, 100%), and deionized (DI) water prior to use. For the nonaqueous electrochemical measurements, lithium perchlorate (LiClO_4_, Acros Organics, 99+%) or tetrabutylammonium perchlorate (Bu_4_NClO_4_, Fluka, >98%) was used in acetonitrile (ACN, VWR, 100%) with methanol (VWR, 100%) as the sacrificial hole scavenger. For the aqueous experiments, sodium sulfate (Na_2_SO_4_, VWR) or sodium sulfite (Na_2_SO_3_, Sigma-Aldrich, ≥98%) was the electrolyte. For the ion chromatography related electrochemical measurements, potassium chloride (KCl, VWR) solution was used as the electrolyte to avoid the interference between sodium and lithium. Before the electrochemical measurements, the nonaqueous LiClO_4_ and Bu_4_NClO_4_ electrolyte solutions were dried using molecular sieves (3 Å, 3–5 mm, Alfa Aesar).

### Preparation of TiO_2_ Layers

The FTO-coated glass substrates were sonicated in acetone, 2-propanol, and DI water for 5–5 min prior to use. The P25 TiO_2_ nanoparticles were spray-coated on preheated substrates (at 140 °C) from an ethanol-based suspension (5 mg cm^–3^). During the spray-coating process, the electrodes were masked to have an exposed geometrical area of 1 cm^2^. The same loading was achieved by using the same number of spray-coating cycles (at 60 cycles, ∼80 μg cm^–2^). After the spray-coating process, the electrodes were heat-treated in a tube furnace at 400 °C for 2 h in an air, argon, or hydrogen atmosphere. This is the highest temperature that can be used in a hydrogen atmosphere without reducing the underlying FTO to metallic Sn.^20^ Furthermore, this temperature is sufficient to substantially increase the trap state density of w-TiO_2_ and form b-TiO_2_.

### Materials Characterization

X-ray diffraction (XRD) measurements were carried out with a Bruker D8 Advance instrument with a Cu K_α_ (λ = 1.5418 Å) X-ray source in the 20–60° 2 theta range with a scan speed of 0.25° min^–1^. For XRD measurements, thicker TiO_2_ films were prepared on glass substrates. Rietveld refinement of the recorded patterns was performed with the GSAS-II software package.^37^ X-ray photoelectron spectroscopy (XPS) was performed with a SPECS instrument equipped with a PHOIBOS 150 MCD 9 hemispherical analyzer. The analyzer was in fixed analyzer transmission mode with a 20 eV pass energy. The Al Kα radiation (*h*ν = 1486.6 eV) of a dual-anode X-ray gun was used as an excitation source and operated at 150 W power. A total of 10 scans were averaged to get a single high-resolution spectrum. The adventitious carbon peak was set at 284.8 eV in all cases. For spectrum evaluation, the CasaXPS commercial software package was used. Ultraviolet photoelectron spectroscopy (UPS) was performed with a He (I) excitation (21.22 eV) source. There was 10 V of external bias applied to the samples to accelerate secondary electrons to the analyzer. The work function of the samples (φ) was determined by φ = *hν* – (*E*_cut-off_ – *E*_F_), where *h*ν is the photon energy of He(I) (21.22 eV), *E*_cutoff_ is the secondary electron cutoff energy, and *E*_F_ is the Fermi energy. A work function value of 4.50 eV for the w-TiO_2_ and 4.53 eV for the b-TiO_2_ was determined. The binding energy scale was corrected (with 0.25 eV for b-TiO_2_ and 0.77 eV for w-TiO_2_, respectively) so that the rise of the UPS signals is at 0 eV, as the sample is perfectly in contact with the analytic chamber of the UPS/XPS machine and the detector. For certain samples, Ar^+^ bombardment was carried out with 2 keV energy for 5 min with a 5.0 μA cm^–2^ sample current in all cases.

### Electrochemical and Spectroelectrochemical Measurements

All electrochemical measurements were carried out with a Biologic VMP-300 type potentiostat/galvanostat in a classical three-electrode setup. The FTO/TiO_2_ electrodes were used as the working electrode, and a platinum foil functioned as the counterelectrode. In nonaqueous media, a Ag/AgCl wire, while in aqueous media a Ag/AgCl wire in 3 mol dm^–3^ NaCl, was used as the reference electrode. All currents were normalized to the geometric surface area of the electrodes. The media for nonaqueous measurements was 1 mol dm^–3^ LiClO_4_ or 1 mol dm^–3^ Bu_4_NClO_4_ in ACN with 5 V/V% methanol, while for the aqueous experiments, 0.1 mol dm^–3^ Na_2_SO_4_ or 0.1 mol dm^–3^ Na_2_SO_3_ solutions were used. The potential of the Ag/AgCl pseudoreference electrode was calibrated before and after the experiments, by measuring the formal potential of the ferrocene/ferrocenium redox couple (0.005 mol dm^–3^ ferrocene (98%, Aldrich) in 1 mol dm^–3^ LiClO_4_ in ACN at multiple sweep rates). The formal potential was determined by cyclic voltammetry (+0.215 V vs Ag/AgCl).

PEC experiments were carried out using a UV lamp (Hamamatsu L8251) or a solar simulator (Newport LCS-100, AM1.5) as the light source with a power density of 100 mW cm^–2^. In all cases, the sweep rate was kept at 2 mV s^–1^, while the light-chopping frequency was 0.10 Hz.

For the Mott–Schottky analysis, impedance spectra were recorded at different potential values in the 100 kHz to 0.1 Hz frequency range, using a sinusoidal excitation signal (10 mV RMS amplitude). Before each measurement, a pretreatment step was applied at the given potential for 2 min. The impedance spectra were measured starting from 0.0 V vs Ag/AgCl and toward more negative potentials.

For the spectroelectrochemical experiments and recording UV–vis spectra of different TiO_2_ electrodes, an Agilent 8453 UV– visible diode array spectrophotometer was used in the 300–1100 nm wavelength range. During the cyclic voltammetric measurements, a sweep rate of 1 mV s^–1^ was applied.

In situ Raman spectroelectrochemical measurements were carried out by a SENTERRA II Compact Raman microscope, using a 532 nm laser excitation wavelength with a laser power of 2.5 mW. The experiment was performed in an ECC-Opto-Std electrochemical cell (EL-CELL GmbH) equipped with a sapphire window, using a potentiostat/galvanostat (Metrohm Autolab PGSTAT204). The spectra were recorded after a 100 s potentiostatic conditioning step at the given potential. In this special setup, a platinum mesh covered with TiO_2_ functioned as the working electrode.

IPCE curves were measured using a Newport Quantum Efficiency Measurement System (QEPVSI-B) in a single-compartment, three-electrode quartz electrochemical cell, in the wavelength range of 300–600 nm (Δλ = 10 nm step size), at the potential of 0.60 V (vs Ag/AgCl).

### Kelvin Probe Microscopy

The measurements were performed using a KP Technology APS04 instrument. Surface photovoltage spectroscopy (SPS) measurements were carried out to determine the lowest wavelength that can create electron–hole pairs in the semiconductor. During the SPS measurements, the sample surface was illuminated with different light energies in the near-infrared and visible regime starting from 1000 to 400 nm. Before measurements, the Fermi level of the gold-alloy-coated tip was determined by measuring the Fermi level of a reference Ag target (*E*_Fermi,Au tip_ = 4.70 eV).

## Supporting Information Available

Additional UV–vis and Raman spectra, spectroelectrochemistry, photovoltammetry, impedance spectroscopy, Mott–Schottky plots, cyclic and linear voltammetry, XPS and UPS measurements, ion chromatography, and surface photovoltage spectroscopy data are shown (PDF)
