# Supplementary material for: The Mystery of Black TiO2: Insights from Combined Surface Science and In Situ Electrochemical Methods
Source: ACS Mater Au. 2021 Aug 3;1(2):157–68. doi: 10.1021/acsmaterialsau.1c00020 (PMC8609907; doi:10.1021/acsmaterialsau.1c00020)
Supplement: Supplementary file 1 — mg1c00020_si_001.pdf [file mg1c00020_si_001.pdf]

Supporting Information

The Mystery of Black TiO_2: Insights from Combined Surface Science and in situ Electrochemical Methods

Ádám Balog, Gergely F. Samu, Szabolcs Pető, Csaba Janáky*

Department of Physical Chemistry and Materials Science, Interdisciplinary Excellence Centre, University of Szeged, Aradi Square 1, Szeged, H-6720, Hungary

Additional figures and tables

Table S1. The observations gathered from the literature and their possible explanations. These can be sorted into two groups (red and green). The sections in red signal observations that conflicts a previous green ones.

Observations	Explanations
Darker color, increased visible and near infrared absorption of b-TiO_2	Narrower bandgap because of the synergistic presence of oxygen vacancies and surface disorders
b-TiO_2 shows disordered surface shell with a crystalline core, decreased intensity of the X-ray diffractions, broader Raman peaks, the presence of Ti^{3+} ions in the XPS spectrum	Defects are introduced in the lattice of b-TiO_2 during the heat treatment in hydrogen; oxygen vacancies are present
b-TiO_2 has enhanced PEC performances, less negative photocurrent saturation potential, improved photoactivity in the UV region	Dopant-free material; there are no charge carrier recombination centers; Fermi level shift toward the conduction band; increased degree of band bending; efficient charge separation and transportation
b-TiO_2 has increased donor density and electrical conductivity	High density of oxygen vacancies which behave as electron donors
b-TiO_2 has much better capacitive properties	Enhanced carrier density and increased density of hydroxyl groups on the surface
The outermost layer in b-TiO_2 consists of a disordered Ti_2O_3 shell	The narrower bandgap is due to the formation of Ti_2O_3 on the surface which is another semiconductor with smaller bandgap energy; the existence of this compound explains why Ti^{3+} is so stable in b-TiO_2
The presence of trap states is disadvantageous, they should be passivated to get better PEC behavior	These states accelerate the charge carrier recombination; this pathway can be eliminated by electrochemical reduction of TiO_2 in the presence of Li^+ ions
w-TiO_2 shows the best photocatalytic activity	During the oxidation of b-TiO_2, the amorphous structure becomes completely crystalline which results in the depletion of trapped charge carriers

Characterization of the different TiO_2 electrodes

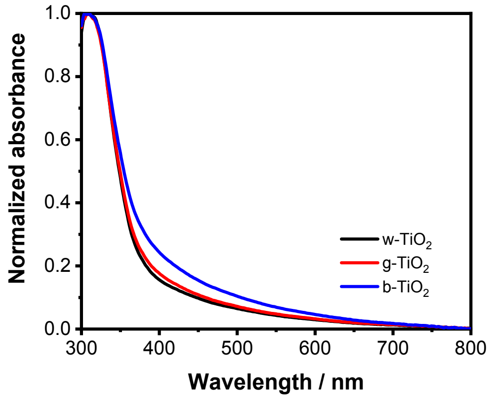

Figure S1. UV-vis absorbance spectra of w-, g- and b-TiO_2 electrodes.

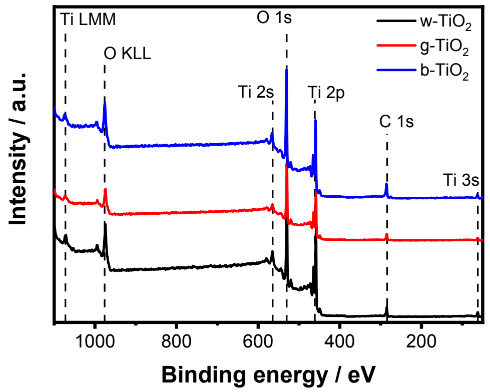

Figure S2. XPS survey spectra of w-, g- and b-TiO_2 electrodes.

XPS fitting procedure:

The C 1s region was fitted with 4 components: (i) C-C, C-H set at 284.8 eV, (ii) C-OH, C-O-C set 1.4 eV above the main peak, (iii) O-C-O set 2.7 eV above the main peak, and (iv) O=C-O set 4.3 eV above the main peak. A U2 Tougaard function was used, to correct for the background. In all cases the line shape was a mixture of Gaussian-Lorentzian (GL30). The FWHM of the peaks were set to be identical for these fittings. In the case of Ti 2p region a Shirley background was used. The line shapes were GL(50) in these cases. The FWHM of the $2p_{1/2}$ was set to be approximately 1.6 times the FWHM of the $2p_{3/2}$ peak. This behavior can be often encountered in the case of transition metal oxides. The energy separation of these two peaks was held constant for the fitting with a 5.78 eV value. The O 1s region was also fitted with a Shirley background. In these cases, contribution from the lattice oxide, surface hydroxides was taken into account. The used line shapes were GL(30) for all components. In

these cases, the FWHM of the surface hydroxide species is double than the lattice oxide peak which is characteristic of transition metal oxide surfaces.

UPS fitting procedure:

In the case of fitting the UPS spectra a Tougaard background was used. The line shapes of the components used for the fitting was GL(20). A summary of the binding energies and FWHM values can be found in Table S2.

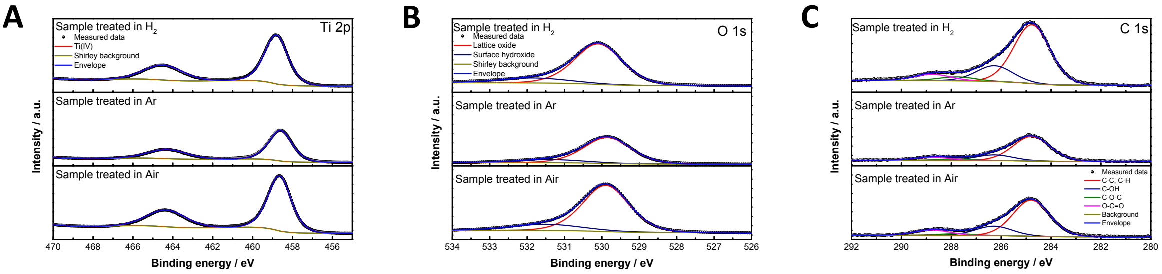

Figure S3. High resolution XPS spectra of the (A) Ti 2p, (B) O 1s and (C) C 1s region of w-, g- and b-TiO_2 electrodes.

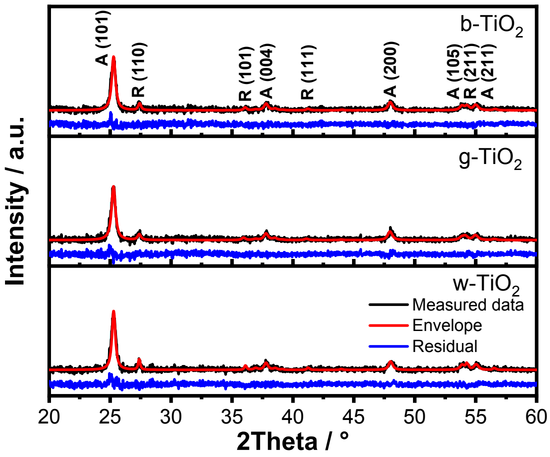

Figure S4. Rietveld refined XRD patterns of w-, g- and b-TiO_2 electrodes.

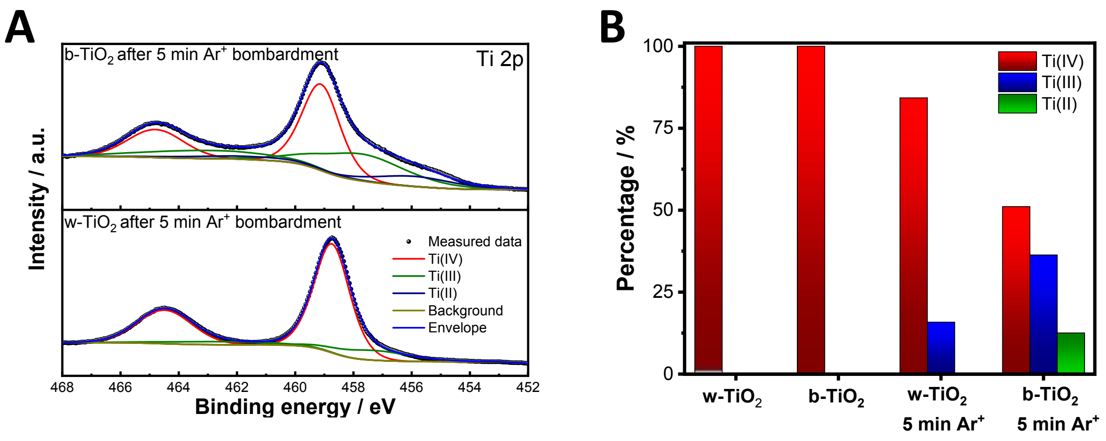

Figure S5. **A:** High resolution XPS spectra of Ti 2p region showing the effect of Ar^+ bombardment on the $2p_{3/2}$ peak position of Ti(IV). **B:** The distribution of different chemical Ti-species on the sample surface before and after Ar^+ bombardment.

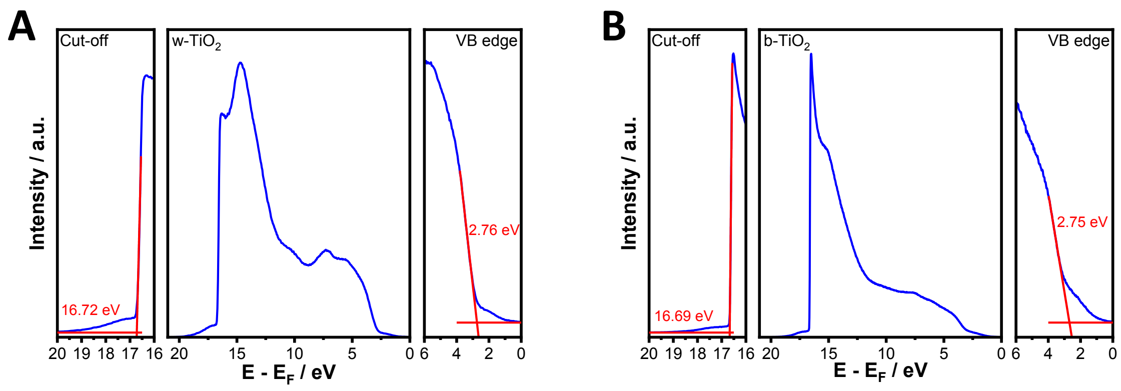

Figure S6. Raw He(I) ultraviolet photoelectron spectra of (A) w- and (B) b-TiO_2 samples. The spectra were acquired with a 10.0 V electrical bias applied to the sample.

Table S2. Summary of the different states used for fitting the UPS data together with the comparison with binding energy values reported in the literature.[1,2]

		Black-TiO_2		White-TiO_2		Anatase powder from ref 1		Molecular beam deposited TiO_2 from ref 2	
Used notation	Assigned states	Binding energy / eV	FWHM	Binding energy / eV	FWHM	Binding energy / eV	FWHM	Binding energy / eV	FWHM
State I	σ bonded state MO with A1g symmetry (surface OH groups)	9.68	2.0	9.55	1.9	10.80	1.2	10.48	-
State II	σ bonded state MO with Eg symmetry	7.70	2.4	7.24	2.4	8.10	2.8	7.28	-
State III	π bonded state (Ti 3d and O 2p) MO with T2g symmetry	5.76	2.1	5.32	2.1	6.00	1.9	5.21	-
State IV	O 2p nonbondin g state	4.22	1.5	2.93	1.4	4.85	1.3	4.24	-
State V	Surface defects	2.64	1.9	1.95	1.8	-	-	-	-

Photoelectrochemistry in aqueous media

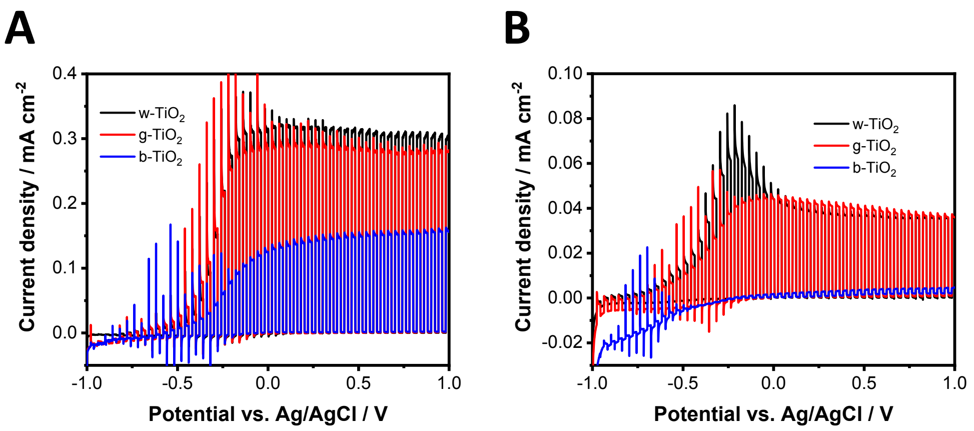

Figure S7. Photovoltammograms of w-, g- and b-TiO_2 electrodes. The measurement was recorded in argon-saturated 0.1 mol dm^{-3} Na_2SO_4 electrolyte in water, using (A) a UV-lamp and (B) a solar simulator (AM1.5) as the light source operated at 100 mW cm^{-2}. The sweep rate was kept at 2 mV s^{-1}, while the light-chopping frequency was 0.10 Hz.

Discussion of EIS results in aqueous media

The shape of the Bode plots changed as a function of the applied potential in all cases, but the potential range where this change occurred was different. In the case of w-TiO_2, the disappearance of the phase angle minimum at higher frequencies is not visible because it contains a negligible amount of Ti(III) states (Figure S8A). In the case of the g- and b-TiO_2 as the number of defect sites increases, this phase angle minimum becomes more obvious and the potential where the phase angle decay starts shifts to less negative values (Figure S8B and C). This change starts from -0.4 V in the case of the w-TiO_2, -0.3 V for the g- one, and -0.2 V for b-TiO_2.

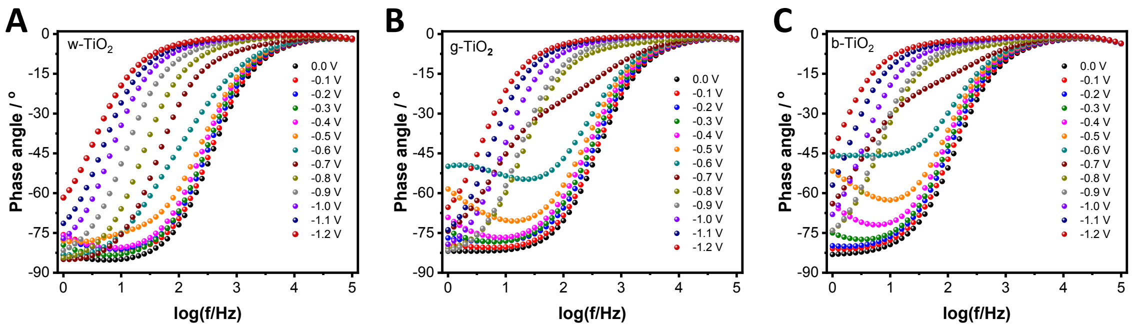

Figure S8. Electrochemical impedance spectroscopic measurements of annealed electrodes: Bode plots of (A) w-, (B) g- and (C) b-TiO_2 electrodes recorded in argon-saturated 0.1 mol dm^{-3} Na_2SO_4 electrolyte in water at different potentials.

Photoelectrochemistry in nonaqueous media

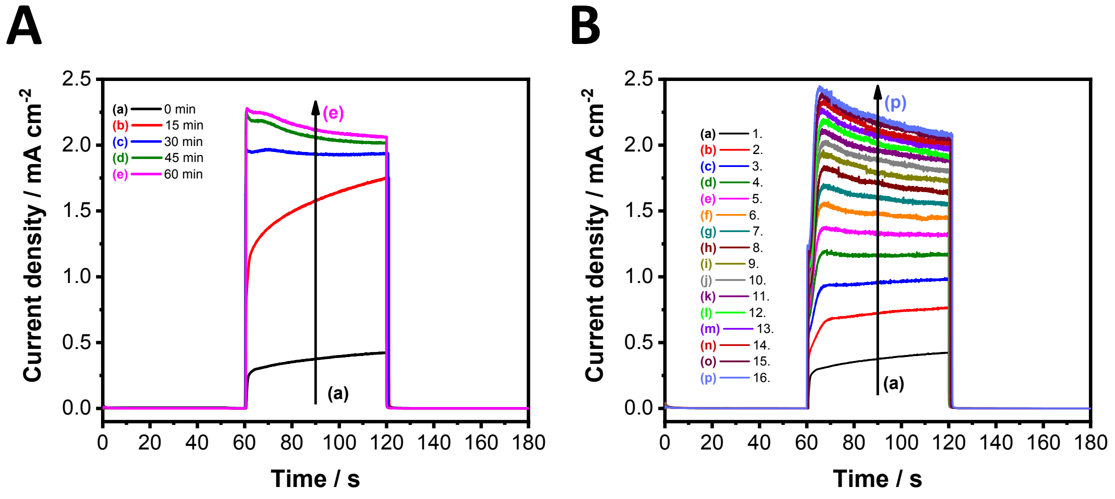

Figure S9. (A) Potentiostatic measurements at +0.6 V in every 15 minutes after the immersion of the electrode in the solution and (B) potentiostatic measurements at +0.6 V after a pretreatment at -0.4 V for a minute (the numbers indicate the ordinal number of measurements). The experiments were recorded for b-TiO_2 electrodes in argon-saturated 1 mol dm^{-3} $LiClO_4$ electrolyte in acetonitrile, using a UV-lamp as the light source operated at 100 mW cm^{-2}.

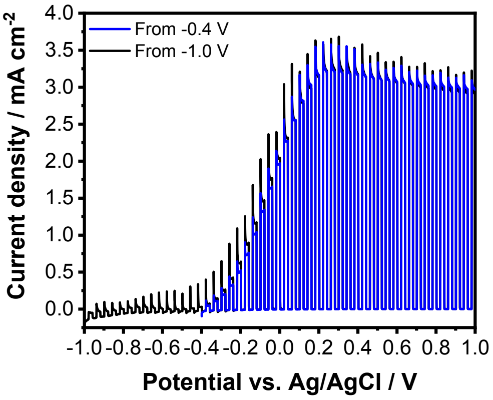

Figure S10. Photovoltammograms of w-TiO_2 starting from -0.4 and -1.0 V. The measurements were recorded in argon-saturated 1 mol dm^{-3} $LiClO_4$ electrolyte in acetonitrile, using a UV-lamp as the light source operated at 100 mW cm^{-2}. The sweep rate was kept at 2 mV s^{-1}, while the light-chopping frequency was 0.10 Hz.

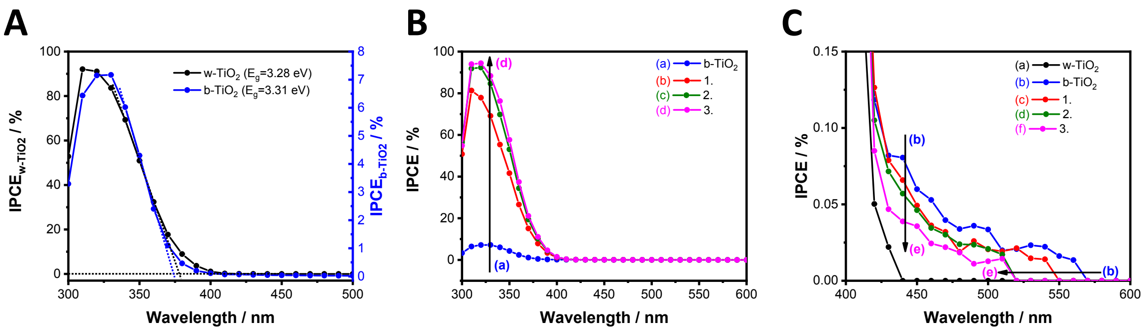

Figure S11. IPCE measurements: (A) bandgap determination for as-synthesized w- and b-TiO_2, (B) effect of passivation for the IPCE values of b-TiO_2 after three consecutive photoelectrochemical measurements started from -1.0 V and (C) IPCE values in the magnified visible region. The experiments were recorded in argon-saturated 1 mol dm^{-3} $LiClO_4$ electrolyte in acetonitrile at 0.6 V.

Effect of trap state passivation on the surface composition

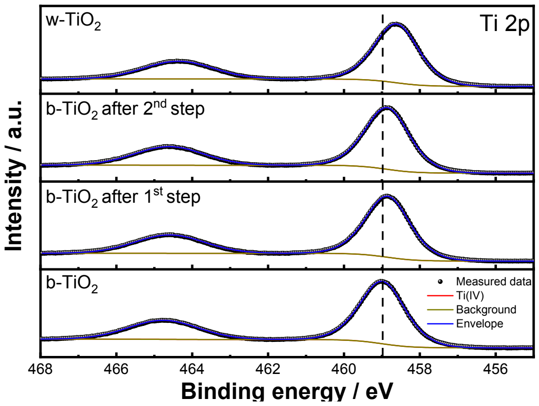

Figure S12. High resolution XPS spectra of Ti 2p region showing the effect of the Li-passivation step on the $2p_{3/2}$ peak position of Ti(IV). The dashed line represents the Ti(IV) peak in b-TiO_2.

Table S3. Peak position of Ti(IV) in w-, b- and trap state passivated b-TiO_2.[3]

Sample	Ti(IV) $2p_{3/2}$ / eV	Ti(IV) $2p_{1/2}$ / eV
b-TiO_2	**458.92 ± 0.08**	**464.71 ± 0.07**
b-TiO_2 after1st step	**458.85**	**464.60**
b-TiO_2 after 2nd step	**458.86**	**464.61**
w-TiO_2	**458.59 ± 0.04**	**464.34 ± 0.04**
Literature w-TiO_2	458.60	464.36
Literature b-TiO_2	459.25	465.00

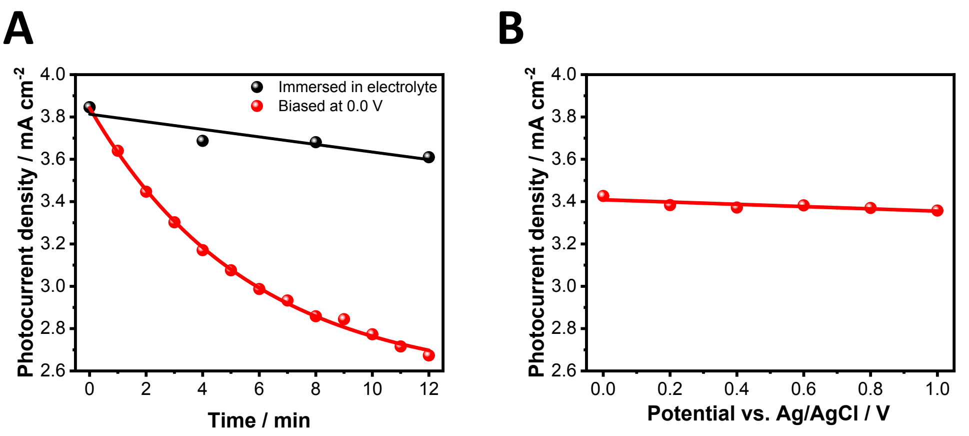

Figure S13. Photocurrents derived from potentiostatic measurements of b-TiO_2 at +0.6 V after the passivation at -1.0 V for a minute. The current values were measured (A) after waiting at OCP (black) and after a pretreatment at 0.0 V (red) for different times and (B) using a one-minute pretreatment step at different potentials. The experiments were recorded in argon-saturated 1 mol dm^{-3} $LiClO_4$ electrolyte in acetonitrile, using a UV-lamp as the light source operated at 100 mW cm^{-2}.

Detection of incorporated Li^+ with ion-chromatography

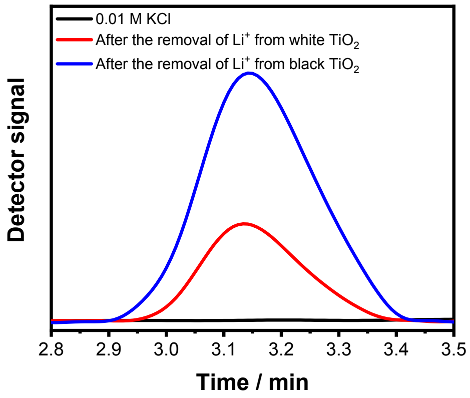

Figure S14. Ion chromatography measurements of pure 0.01 mol dm^{-3} KCl in water, and the same electrolyte after the removal of Li^+ ions from w- and b-TiO_2 at 0.6 V for 30 minutes.

Effect of passivation on the capacitance and donor density

Besides the photocurrent values, the capacitance values can also be changed by the passivation of trap states which was confirmed by the capacitance values determined from cyclic voltammetry (Figure S15). The reason is probable the decrease in the number of Ti(III) states during the measurement in potential range more negative than -0.4 V by the passivation effect of Li^+. To support this statement, Mott-Schottky experiments were carried out (Figure S16) which indicate the decrease in the number of donors. Closer inspection of the potential dependence of the C^{-2} values, shows that after an initial increase from -0.4 V an abrupt decrease at the flatband potential is observed.

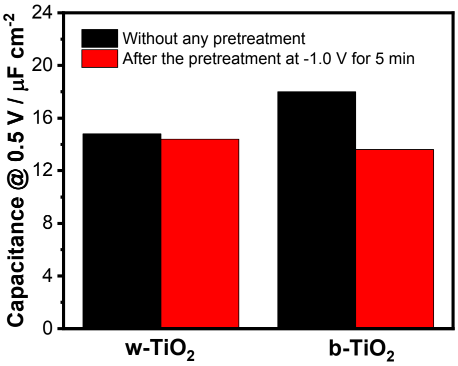

Figure S15. Capacitance values at 0.5 V of w- and b-TiO_2 before and after the pretreatment at -1.0 V for 5 minutes. The values were determined from cyclic voltammetry between 0.0 and 1.0 V in argon-saturated 1 mol dm^{-3} $LiClO_4$ electrolyte in acetonitrile with a sweep rate of 100 mV s^{-1}.

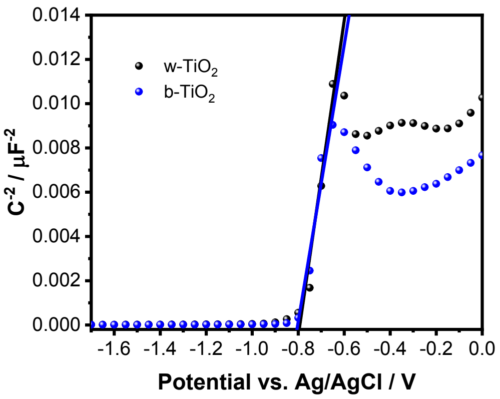

Figure S16. Mott−Schottky plot at 1 kHz frequency of w- and b-TiO_2 electrodes in argon-saturated 1 mol dm^{-3} $LiClO_4$ electrolyte in acetonitrile.

Note that by plotting the C^{-2} on the y axis in the Mott-Schottky plot, a larger value translates to a lower capacitance. The decrease in the capacitance is observable in both cases, however, b-TiO_2 shows larger difference between the first and second scan (Figure S17). This is consistent with the previous results that more Li^+ are needed to cover the increased amount of defects. During the initial phase of these measurements, the applied potential can even start depopulating the Ti(III) states. These results also suggest that the depopulation step is slower than the passivation process.

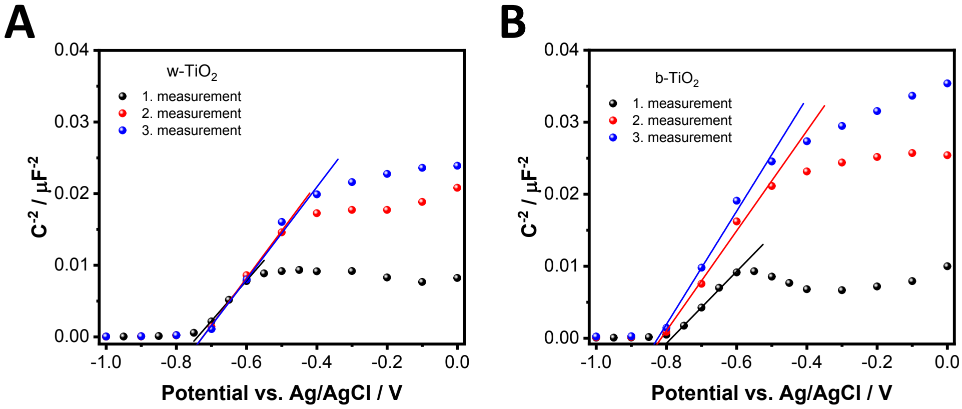

Figure S17. Mott-Schottky plots of (A) w- and (B) b-TiO_2 electrodes at 1 kHz frequency in argon-saturated 1 mol dm^{-3} $LiClO_4$ electrolyte in acetonitrile.

UV-vis spectroelectrochemistry in non-aqueous media

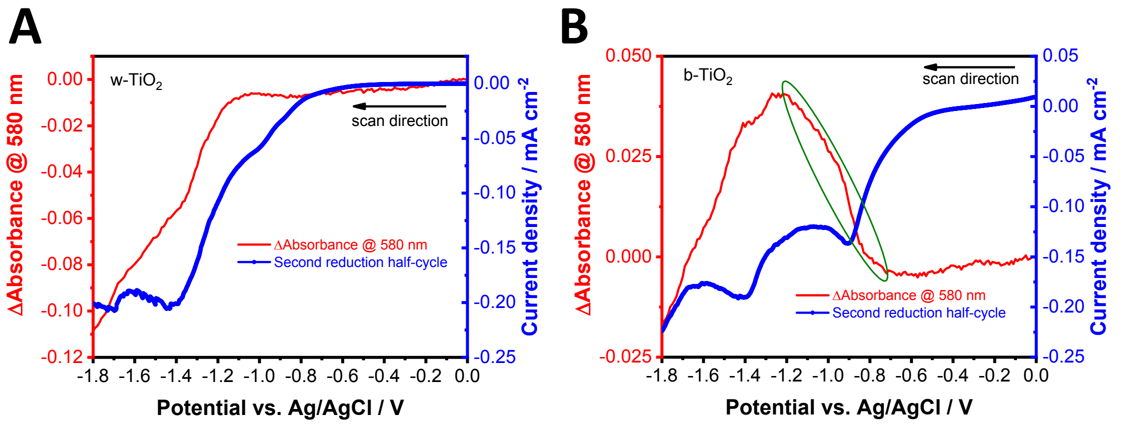

Figure S18. Spectroelectrochemical data, recorded for w- and b-TiO_2 electrodes. The second reduction half-cycles of (A) w- and (B) b-TiO_2 are plotted together with the absorbance change at 580 nm. The experiments were recorded in oxygen-saturated 1 mol dm^{-3} $LiClO_4$ electrolyte in acetonitrile with a sweep rate of 1 mV s^{-1}. The green circles highlight the change of the absorbance during the electron injection into the trap states.

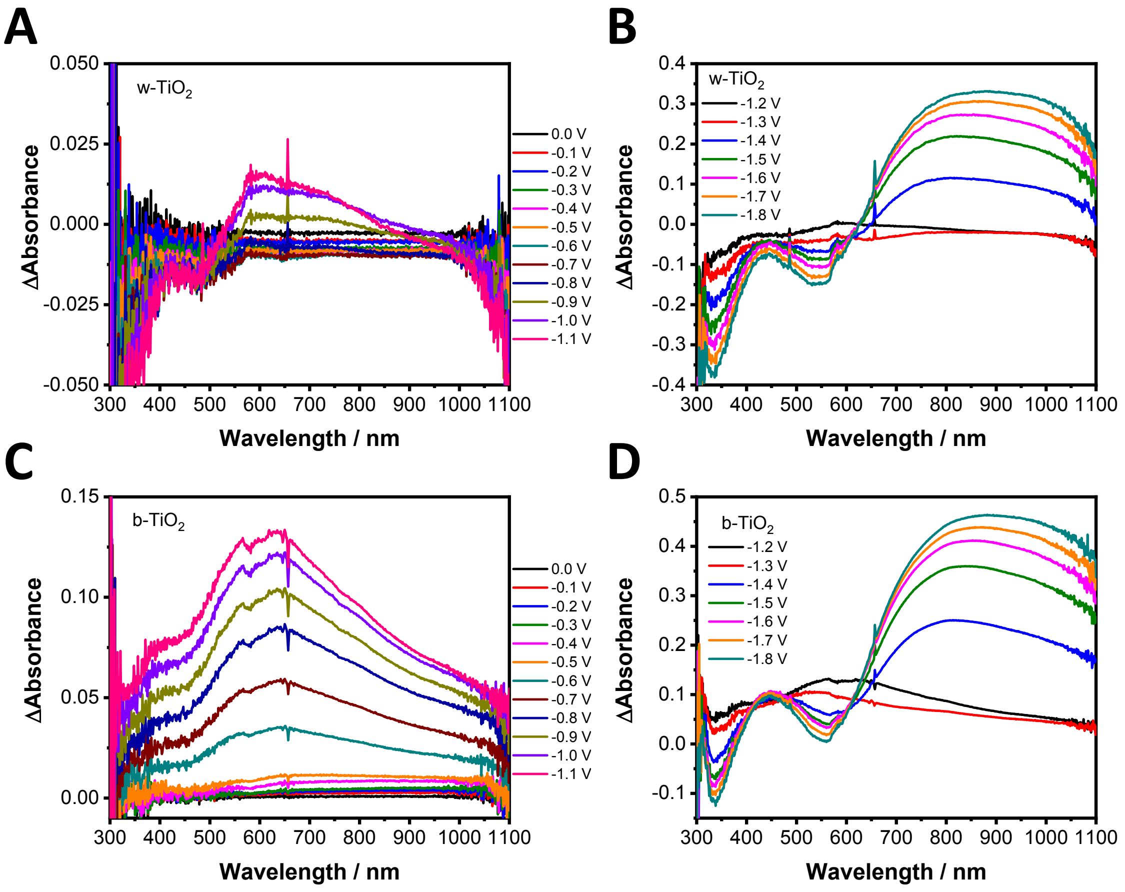

Figure S19. UV−vis absorbance difference spectra at different potentials of (A, B) w- and (C, D) b-TiO_2 electrodes. The experiments were recorded in oxygen-saturated 1 mol dm^{-3} $LiClO_4$ electrolyte in acetonitrile with a sweep rate of 1 mV s^{-1}.

Raman spectroelectrochemistry in non-aqueous media

The Raman spectrum of w-TiO_2 at open circuit potential (Figure S20B) shows peaks at 144, 197 and 398 cm^{-1} which can be attributed to the O-Ti-O bending type vibrations, while bands at 518 and 639 cm^{-1} corresponds to the Ti-O bond stretching type vibrations of anatase.[4] The weak band located at 452 cm^{-1} is characteristic of rutile phase,[5] while the other peaks are due to the electrolyte.[6,7] b-TiO_2 shows identical Raman-modes, that are slightly broadened (Figure S20C). In addition, further new weak and broad bands appear which cannot be assigned to any of the three polymorphs of TiO_2. This indicates the structural changes after hydrogen treatment, resulting in structural disorder that can activate otherwise Raman-forbidden modes.[8]

Until the onset of the bulk reduction of TiO_2 (~-1.2 V vs. Ag/AgCl), the Raman spectra were unchanged in the case of both samples (Figure 8A and S20C), however, at more negative potentials, several changes can be detected (Figure 8B and S20D). The cyclic voltammograms are shown here only to help the identification of the processes as a function of the applied potential (Figure S20A). The position of the peak at 518 cm^{-1} shifted to 531 cm^{-1} and the band at 639 cm^{-1} is also shifted to 630 cm^{-1}. Several new bands also appeared: a band at 559 cm^{-1}, another broad band structure around 300 cm^{-1} with a peak at 357 cm^{-1} and two bands in the low-wavenumber region at 166 and 233 cm^{-1}.[9,10] In addition, the intensity of the peak at 144 cm^{-1} decreased and finally disappeared completely.[11]

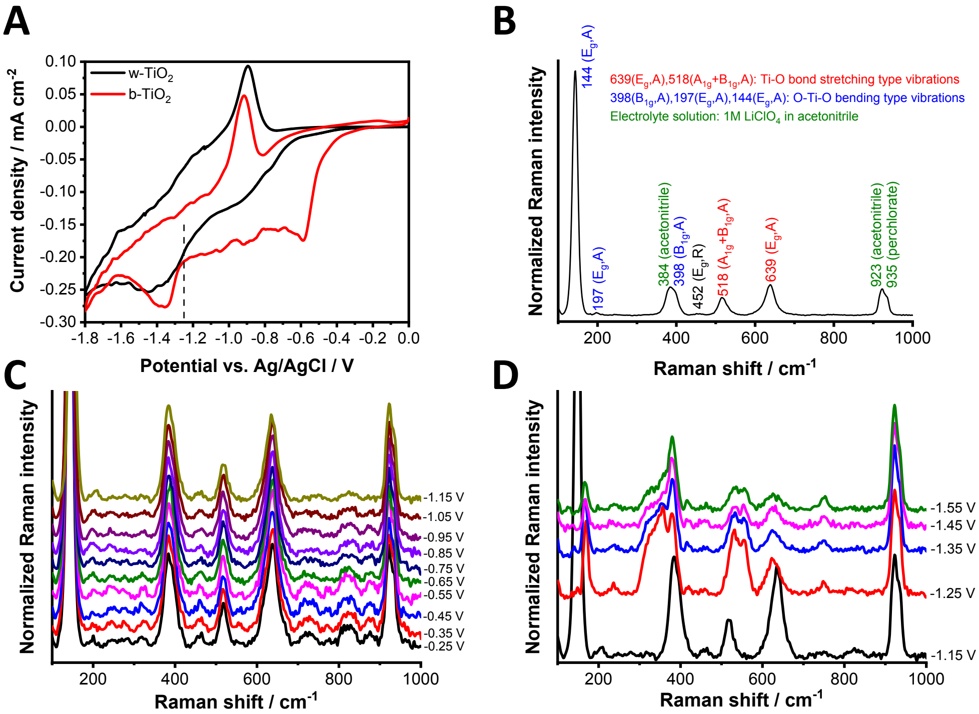

Figure S20. (A) Cyclic voltammograms of w- and b-TiO_2; (B) Raman spectrum of w-TiO_2 at open circuit potential; in situ Raman spectroelectrochemistry of b-TiO_2 in the range between (C) -0.25 to -1.15 V and (D) -1.15 to -1.55 V. The experiments were recorded in 1 mol dm^{-3} $LiClO_4$ electrolyte in acetonitrile.

Surface photovoltage spectroscopy

Based on the SPS measurements (Figure S21), 1.61 eV is enough to excite charge carriers in w-TiO_2, while this energy is 1.40 eV in the case of b-TiO_2. These values are related to the excitation from the edge of the VB tail to the CB. This sensitive method proves that we can generate charge carriers with low photon energies, however, we cannot extract them as photocurrent during the PEC measurements.

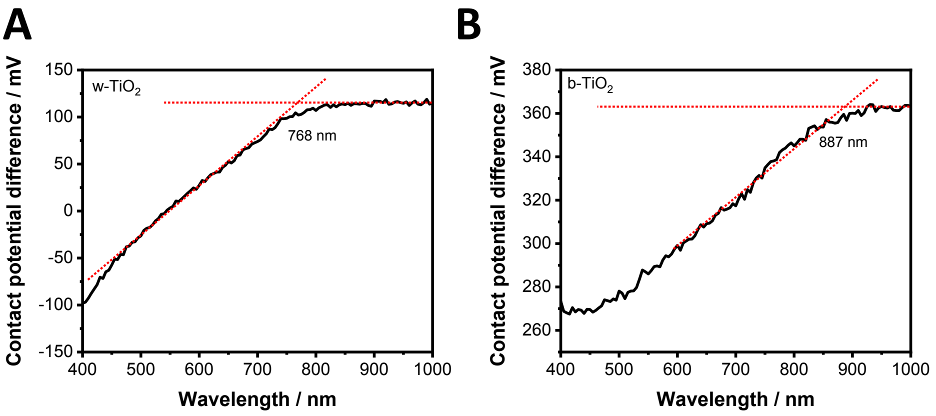

Figure S21. Surface photovoltage spectroscopy measurements of (A) w- and (B) b-TiO_2 electrodes.

Photoelectrochemical response in diluted electrolytes

We also carried out similar PEC measurements in more dilute electrolytes (Figure S22) to exclude effects from the high ionic strength used. Similar differences were found between the samples: (i) no trap state passivation when Bu_4N^+ is present in the solution, (ii) two observable steps in trap state passivation in Li^+ ion containing media (Figure S22A and C), (iii) the concentration of Li^+ has no effect on the relative photocurrent increase (Figure S22B). The only difference is that higher photocurrents were observed in the media with low ionic strength.

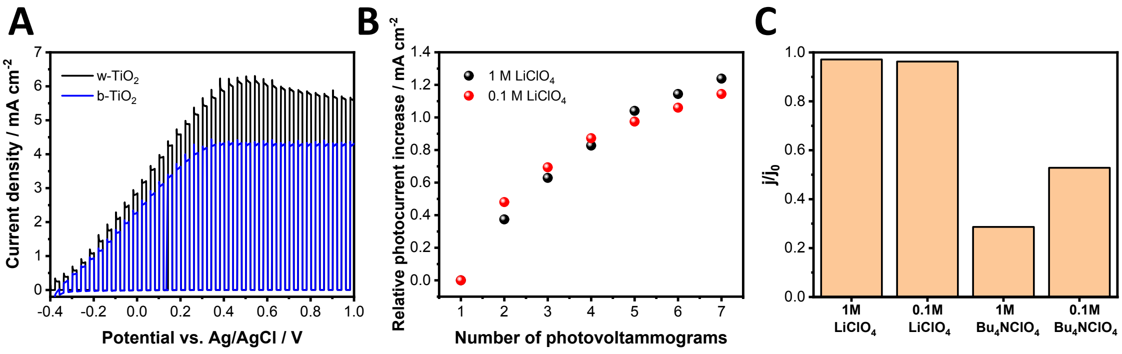

Figure S22. (A) Photovoltammograms of w- and b-TiO_2 electrodes in 0.1 mol dm^{-3} $LiClO_4$ electrolyte in acetonitrile, using a UV-lamp as the light source operated at 100 mW cm^{-2}. The sweep rate was kept at 2 mV s^{-1}, while the light-chopping frequency was 0.10 Hz. (B) Relative photocurrent increase during seven consecutive LSV measurements starting from -0.4 V in the two different $LiClO_4$ electrolytes with different concentrations. (C) Maximum photocurrent achieved with b-TiO_2 (after the -1.0 V treatment) (j) divided by the photocurrent of the white one (j_0) in a given electrolyte (the current values are related to 0.6 V).

Supplementary references

(1) Maheu, C.; Cardenas, L.; Puzenat, E.; Afanasiev, P.; Geantet, C. UPS and UV Spectroscopies Combined to Position the Energy Levels of TiO_2 Anatase and Rutile Nanopowders. *Phys. Chem. Chem. Phys.* **2018**, *20* (40), 25629–25637.

(2) Fleming, L.; Fulton, C. C.; Lucovsky, G.; Rowe, J. E.; Ulrich, M. D.; Lüning, J. Local Bonding Analysis of the Valence and Conduction Band Features of TiO_2. *J. Appl. Phys.* **2007**, *102* (3).

(3) Chen, S.; Xiao, Y.; Wang, Y.; Hu, Z.; Zhao, H.; Xie, W. A Facile Approach to Prepare Black TiO_2 with Oxygen Vacancy for Enhancing Photocatalytic Activity. *Nanomaterials* **2018**, *8* (4), 1–16.

(4) Tompsett, G. A.; Bowmaker, G. A.; Cooney, R. P.; Metson, J. B.; Rodgers, K. A.; Seakins, J. M. The Raman Spectrum of Brookite, TiO_2 (Pbca, Z = 8). *J. Raman Spectrosc.* **1995**, *26* (1), 57–62.

(5) Cheng, H.; Ma, J.; Zhao, Z.; Qi, L. Hydrothermal Preparation of Uniform Nanosize Rutile and Anatase Particles. *Chem. Mater.* **1995**, *7* (4), 663–671.

(6) Costa, L. T.; Siqueira, L. J. A.; Nicolau, B. G.; Ribeiro, M. C. C. Raman Spectra of Polymer Electrolytes Based on Poly(Ethylene Glycol) Dimethyl Ether, Lithium Perchlorate, and the Ionic Liquid 1-Butyl-3-Methylimidazolium Hexafluorophosphate. *Vib. Spectrosc.* **2010**, *54* (2), 155–158.

(7) Deàk, J. C.; Iwaki, L. K.; Dlott, D. D. Vibrational Energy Redistribution in Polyatomic Liquids: Ultrafast IR−Raman Spectroscopy of Acetonitrile. *J. Phys. Chem. A* **1998**, *102* (42), 8193–8201.

(8) Chen, X.; Liu, L.; Yu, P. Y.; Mao, S. S. Increasing Solar Absorption for Photocatalysis with Black Hydrogenated Titanium Dioxide Nanocrystals. *Science (80-.).* **2011**, *331* (6018), 746–750.

(9) Smirnov, M.; Baddour-Hadjean, R. Li Intercalation in TiO_2 Anatase: Raman Spectroscopy and Lattice Dynamic Studies. *J. Chem. Phys.* **2004**, *121* (5), 2348–2355.

(10) Baddour-Hadjean, R.; Bach, S.; Smirnov, M.; Pereira-Ramos, J.-P. Raman Investigation of the Structural Changes in Anatase Li_xTiO_2 upon Electrochemical Lithium Insertion. *J. Raman Spectrosc.* **2004**, *35* (7), 577–585.

(11) Hardwick, L. J.; Holzapfel, M.; Novák, P.; Dupont, L.; Baudrin, E. Electrochemical Lithium Insertion into Anatase-Type TiO_2: An in Situ Raman Microscopy Investigation. *Electrochim. Acta* **2007**, *52* (17), 5357–5367.
